# Fatal attraction of *Caenorhabditis elegans* to predatory fungi through 6-methyl-salicylic acid

**DOI:** 10.1038/s41467-021-25535-1

**Published:** 2021-09-15

**Authors:** Xi Yu, Xiaodi Hu, Maria Pop, Nicole Wernet, Frank Kirschhöfer, Gerald Brenner-Weiß, Julia Keller, Mirko Bunzel, Reinhard Fischer

**Affiliations:** 1 Karlsruhe Institute of Technology (KIT) - South Campus, Institute of Applied Biosciences, Department of Microbiology, Fritz-Haber-Weg 4, Karlsruhe, Germany; 2 Karlsruhe Institute of Technology (KIT) - North Campus, Institute of Functional Interfaces, Department of Bioengineering and Biosystems, Eggenstein Leopoldshafen, Germany; 3 Karlsruhe Institute of Technology (KIT) - South Campus, Institute of Applied Biosciences, Department of Food Chemistry and Phytochemistry, Adenauerring 20 A, Karlsruhe, Germany; 4 Present Address: Shanghai Engineering Research Center of Hadal Science and Technology, College of Marine Sciences, Shanghai Ocean University, Shanghai, China

**Keywords:** Chemical ecology, Cellular microbiology, Fungal biology, Fungal ecology

## Abstract

Salicylic acid is a phenolic phytohormone which controls plant growth and development. A methyl ester (MSA) derivative thereof is volatile and involved in plant-insect or plant-plant communication. Here we show that the nematode-trapping fungus *Duddingtonia flagrans* uses a methyl-salicylic acid isomer, 6-MSA as morphogen for spatiotemporal control of trap formation and as chemoattractant to lure *Caenorhabditis elegans* into fungal colonies. 6-MSA is the product of a polyketide synthase and an intermediate in the biosynthesis of arthrosporols. The polyketide synthase (ArtA), produces 6-MSA in hyphal tips, and is uncoupled from other enzymes required for the conversion of 6-MSA to arthrosporols, which are produced in older hyphae. 6-MSA and arthrosporols both block trap formation. The presence of nematodes inhibits 6-MSA and arthrosporol biosyntheses and thereby enables trap formation. 6-MSA and arthrosporols are thus morphogens with some functions similar to quorum-sensing molecules. We show that 6-MSA is important in interkingdom communication between fungi and nematodes.

## Abstract (web summary)

Methyl salicylate ester is a plant metabolite involved in plant-insect and plant-plant interactions. Here, Yu et al. show that a nematode-trapping fungus has potential to produce a related compound, 6-methyl salicylate, which attracts its prey (nematodes) and modulates spore germination and trap formation in the fungus.

## Introduction

Eukaryotic developmental processes depend on an interplay between internal and external signals which together may lead to massive reprogramming of certain cells. A well-accepted concept in developmental biology is the regulation through gradients of active signaling compounds, called morphogens. Such factors can be transcriptional regulators such as *bicoid* in *Drosophila* or extracellular signals^1,2^.

Filamentous fungi are rather simple organisms with clearly defined developmental pathways to produce different cell types and structures. One well-studied example is the model fungus *Aspergillus nidulans* which produces asexual and sexual reproductive structures^3^. Developmental processes are regulated by external stimuli, e.g., light and depend on complicated genetic programs^4^. There is also evidence for endogenous low-molecular weight compounds which may be involved in the spatial regulation of development. The factors are called PSI factors (precocious sexual inducer) and are oxylipin derivatives^5,6^. Several *A. nidulans* enzymes required for the production of the PSI factors have been identified^7,8^. If secreted chemicals are involved in developmental decisions, they are potential targets for other organisms to interfere or change the normal developmental program.

Low-molecular weight compounds were also discovered in nematode-trapping fungi (NTF) interaction^9,10^. This group of fungi can switch between saprotrophic growth and a predatory lifestyle^11^. One of the best-studied examples is the ascomycete *Arthrobotrys oligospora* and the interaction with *Caenorhabditis elegans*^12,13^. The fungus reproduces with asexual conidiospores and, upon starvation and the presence of nematodes, three-dimensional, adhesive trapping networks are formed^14–16^. Low-molecular weight compounds play important roles in the fungal-worm interaction, where *A. oligospora* responds to *C. elegans*-derived ascarosides^10^. Ascarosides are composed of a dideoxy sugar and a fatty-acid like molecule and are important worm signaling molecules^17,18^. On the other hand, *A. oligospora* produces several volatile compounds to lure nematodes into the traps. These volatiles were identified as 2-methyl-1-butanol, 2,4-dithiapentane, methyl 3-methyl-2-butenoate, and S-methyl thioacetate^9^. Not only ascarosides induce the developmental program of trap formation, but also urea. With this compound some bacteria can induce trap formation in the fungus, which then is able to catch the bacterial enemies, the nematodes^19^.

Whereas the described signaling processes that are important for the organismal interactions, other low-molecular weight compounds, which control development, are arthrosporols^20^. Arthrosporols are sesquiterpenol derivatives combined with an epoxy cyclohexanol moiety (SEC). Three related molecules have been isolated from *A. oligospora*, all of which had a negative effect on conidiospore formation, while one stimulated and one reduced the number of traps. The biosynthesis depends on a polyketide synthase, and a corresponding deletion strain produced more traps suggesting a negative role of arthrosporols on trap formation^20,21^. Sesquiterpenols can thus be considered as morphogens controlling development. The discrepancy between the experiments with the purified compound and the in vivo results with the deletion strain may be explained by the fact that in vivo a blend of different arthrosporols is produced and this mixture represses trap formation. Several other sesquiterpenol derivatives have been described^22,23^. However, nothing was known so far about concentration gradients, which may trigger different developmental decisions or the modulation of morphogen production or action in response to *C. elegans*. If *C. elegans* is present, and the mycelium starved, traps should be formed. Hence, trap formation is regulated by the nutritional status of the mycelium, the developmental competence of the hyphae, and the presence of nematodes.

Here, we analyzed arthrosporol formation in *D. flagrans*, a fungus closely related to *A. oligospora*. As a difference to *A. oligospora, D. flagrans* is able to produce resistant chlamydospores in addition to conidiospores. Chlamydospores are able to pass through the intestinal tract of animals and have therefore been used as biocontrol agents in animal farming (https://www.duddingtonia.com)^24^. We studied trap formation at the molecular and cellular level and found that the spatiotemporal differential expression of the arthrosporol producing polyketide synthase and the tailoring enzymes lead to 6-methyl salicylic acid and to arthrosporol. Both compounds inhibit trap formation, and *C. elegans* blocks the production of the inhibitors and thereby induces trap formation. In addition, 6-methyl salicylic acid is a chemical attractant to lure nematodes into the fungal mycelium.

## Results

### Arthrosporols control trap formation in *D. flagrans*

The genome of *D. flagrans* encodes three different polyketide synthases^15^, one of which with high similarity to a polyketide synthase from *A. oligospora* where it is involved in trap formation^20,21^. In order to explore the regulation of this morphogenetic pathway in *D. flagrans*, we studied the corresponding polyketide synthase gene cluster and named the genes *artA-artE* (arthrosporol), with *artA* encoding the synthase (Supplementary Fig. S1a). *artA-E* gene expression was determined with quantitative real-time PCR using mycelia of *D. flagrans* cultured in liquid medium. Expression started after two days of growth and increased steadily over time. The putative transcription factor gene, *artR*, was always very low expressed. Three adjacent genes were poorly expressed and probably do not belong to the cluster (Supplementary Fig. S1b).

**Fig. 1:**
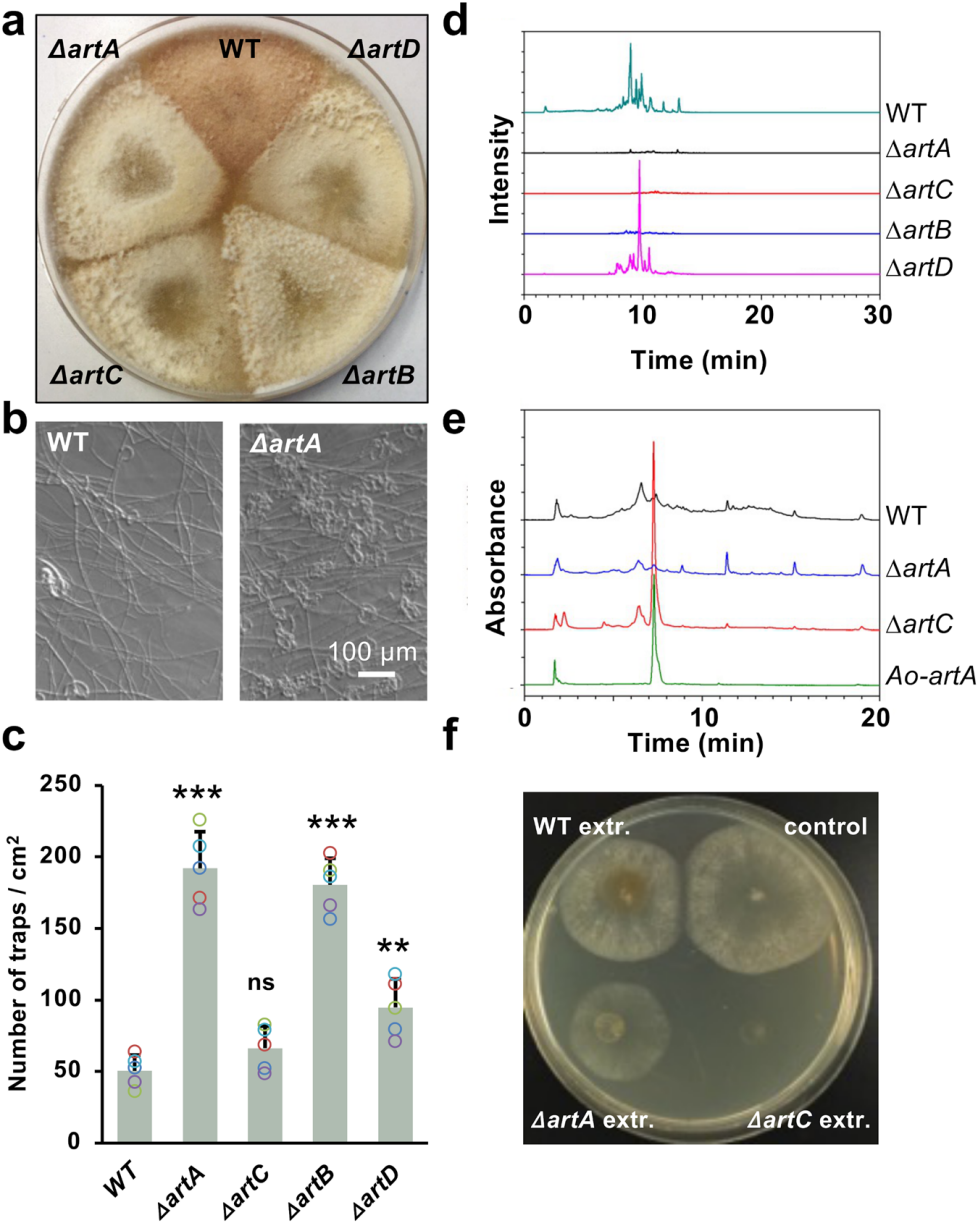
The *artA* gene cluster is required for 6-MSA and arthrosporol biosynthesis. **a** Comparison of colonies of wild-type (WT), a *ΔartA-* (strain SXY05), a *ΔartB-* (strain SXY15)*, a ΔartC-* (strain SXY14), and a *ΔartD-*deletion strain (strain SXY16) after 8 days of growth on PDA agar plates. **b** Trap formation in WT and the *∆artA*-deletion strain (scale bar, 100 µm). **c** Quantification of traps induced by the same number of *C. elegans* N2 in *D. flagrans* strains as indicated (mean ± SD, *n* = 5 biological with 3 technical replicates; asterisks represent significance levels as determined with the unpaired *t*-test compared to WT; *p < 0.05, **p < 0.01, ***p < 0.001; *ΔartA p*-value = 3.36858E-06, *ΔartB p* *=* 0.097611834; *ΔartC p* *=* 9.20668E-07, *ΔartD p* *=* 0.002468371). **d** HPLC-DAD profiles of the extracts of the strains indicated. **e** LC–MS/MS quantification of 6-MSA from culture extracts of the strains indicated. Ao-*artA* (strain SXD01) means *A. oryzae* expressing *artA*. **f** Inhibition of *D. flagrans* by extracts of different strains*. D. flagrans* was point inoculated and 30 µl of the metabolite extracts added. The plate was incubated for 3 days at 28 °C. Source data are provided as a Source Data file.

To decipher the roles of the putative *artA-*cluster genes, *artA-D* were deleted (Supplementary Figs. S2, 3). Whereas wild-type colonies appeared brownish, *∆artA, B, C*, and *D* appeared pale, suggesting a role in pigment formation (Fig. 1a). Next, we studied the effect on trap formation. Wild type produced about 53 traps/cm^2^ whereas the *ΔartA-*deletion strain produced ca. 200 traps/cm^2^ (Fig. 1b, c). In comparison, the *ΔartB* strain produced 183 and the *ΔartD-*deletion mutant 95 traps/cm^2^. The *ΔartC-*deletion mutant produced similar numbers of traps as wild type. The *artA*-deletion strain was re-complemented (strain SXD05) with a wild-type copy of *artA*. In this strain, the trap number was again reduced to the wild-type level (Supplementary Fig. S2c).

**Fig. 2:**
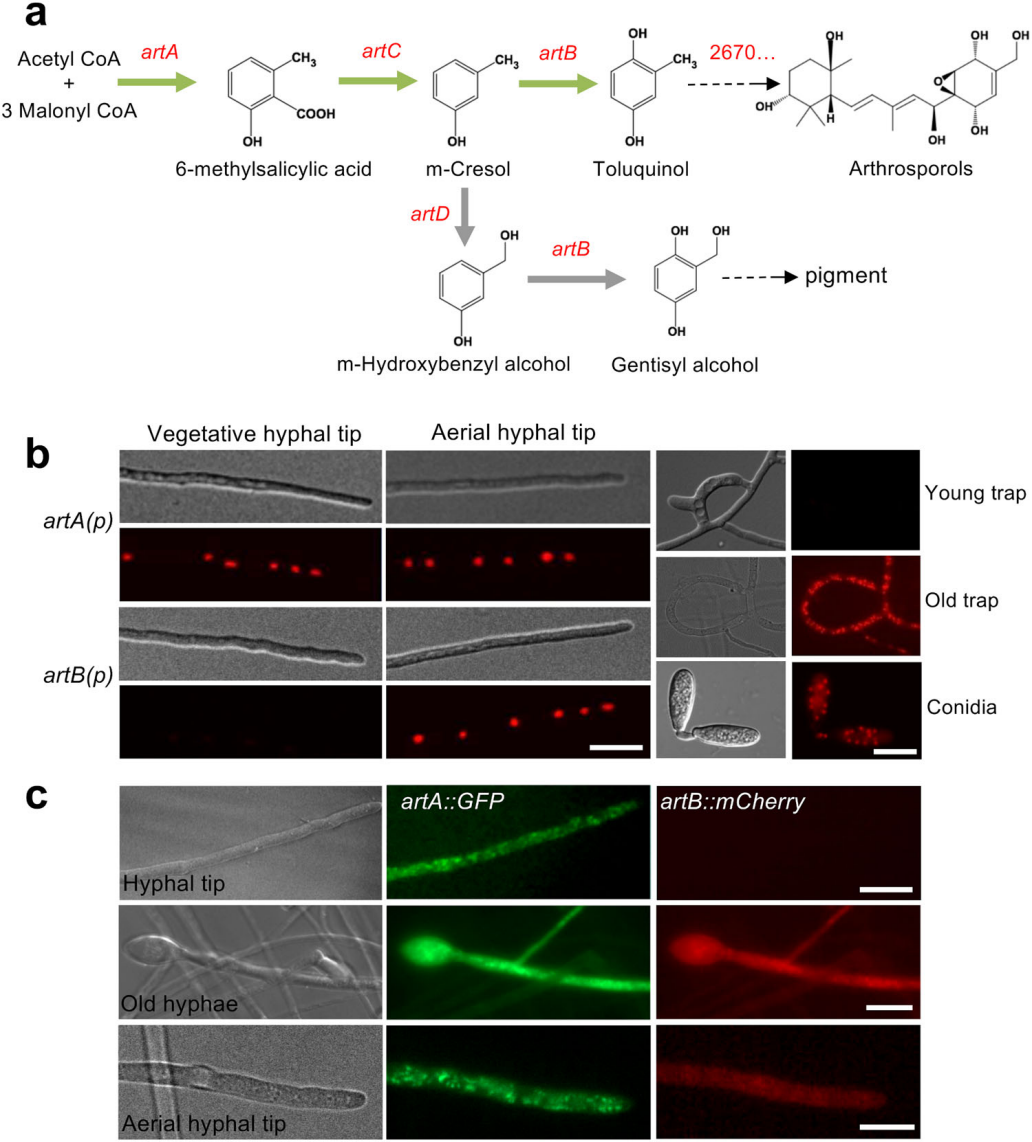
Expression of the polyketide synthase ArtA and the tailoring enzyme ArtB in *D. flagrans*. **a** Proposed pathway for the biosynthesis of 6-MSA, arthrosporols and pigment. Some intermediates were determined in this study (green arrow) and some were deduced from the work in *A. oligospora* (grey arrow). **b** The promoter of *artA* was fused to *h2b* and *mCherry* (strain SXY17) and different hyphal structures were analyzed. Spatiotemporal control of *artA* and *artB* (strain SXY20) expression in vegetative hyphal tips, aerial hyphal tips (old hyphae), new traps, old traps, and conidia. **c** ArtA-GFP and ArtB-mCherry co-expression after integration of the tagging constructs *in locus* (strain SXD53). The scale bars represent 10 µm.

In order to elucidate the *artA*-cluster biosynthetic pathway, we analyzed the culture broths of WT and the *ΔartA-D* deletion strains by high-performance liquid chromatography (HPLC−DAD) and high-resolution LC–MS. Arthrosporol A-C were produced in wild type (monoisotopic mass of 410.2305, A, B and C, Supplementary Fig. S4) and were absent in the *∆artA*, *∆artB*, or the *∆artC*-deletion strains (Fig. 1d). The production of arthrosporols was normal in the *∆artD-*deletion strain. These results suggest that only *artA, artB*, and *artC* are involved in the biosynthesis of arthrosporol A-C (Supplementary Fig. S4), and *artD* is required for pigment biosynthesis. In the ∆*artB*-deletion strain m-cresol (Supplementary Fig. S5), and in the *ΔartC*-deletion strain 6-MSA accumulated, suggesting that 6-MSA is the direct product of ArtA. 6-MSA concentrations were calculated from peak areas in the HPLC profile using 6-MSA standard solutions for calibration. The *artC-*deletion strain produced about 6000 times more 6-MSA than wild type. The fact that 6-MSA was also detected in wild type, suggests that small amounts are produced under natural conditions in the presence of all enzymes. 6-MSA production by ArtA was proven by heterologous expression of ArtA in *Aspergillus oryzae* (strain SXD01) (Fig. 1e, Supplementary Fig. S7)^25^. The obtained results led us to propose a shared biosynthetic pathway for arthrosporols and pigments in *D. flagrans* (Fig. 2a), which is in agreement with the proposed pathway in *A. oligospora*^21,26^.

Next, we tested the effect of extracts of wild type and the different deletion strains on growth and trap formation and found that extracts of the *∆artC-*deletion strain severely reduced hyphal growth (Fig. 1f, Supplementary Fig. S6a). In addition, conidia and chlamydospores rarely germinated even after several days of cultivation. Diluted extracts of the *∆artC-*deletion strain lowered the germination ratio of spores and led to unhealthy hyphae (Supplementary Fig. S6a). The same effects were observed with extracts from the *A. oryzae artA*-expressing strain suggesting that 6-MSA also inhibits trap formation. In addition, germination of spores was blocked, and trap formation gradually inhibited with increasing concentrations of 6-MSA (Supplementary Fig. S6b, c). Extracts of a *D. flagrans* ∆*artC*-deletion strain also inhibited growth of *Alternaria alternata*, whereas extracts of a ∆*artA*-deletion strain did not, showing the broad action of 6-MSA on fungi (Supplementary Fig. S6d).

### The *artA* gene cluster genes are differentially expressed in time and space

The next question was, if the biological activities of arthrosporol and 6-MSA were of any biological meaning. 6-MSA should only be an endogenous intermediate and quickly converted to arthrosporols if all enzymes were expressed (Fig. 2a). In addition, if arthrosporols inhibit trap formation, but are produced early during hyphal growth, the question raises, if and how nematodes may interfere with the production of arthrosporols. Quantitative real-time PCR analyses revealed an increase of transcript abundance with time, but these experiments did not allow any spatial resolution (Supplementary Fig. S1b). Therefore, we developed an assay to monitor the expression of the *artA-C* genes in time and space. To this end, the promoter of the gene of interest was fused with the *h2b* (histone 2 b) gene and with *mCherry*. If a promoter is active, the fusion protein should be concentrated in nuclei. This is very easily distinguished from possible autofluorescence of older hyphae and should be easy to quantify. For *artA*, the strongest fluorescent signals were found in the nuclei in hyphal tips (Fig. 2b). To show that the accumulated fluorescent proteins reflect continuous, active transcription and translation, we bleached the hyphae and observed recovery after 14 min (Supplementary Fig. S8). A similar reporter construct was constructed for *artB* and *artC* (Fig. 2b, Supplementary Fig. S9). In contrast to *artA*, no fluorescent signal was obtained in hyphal tips and young hyphae. However, in older substrate- and arial hyphae, which are produced after some time, fluorescent signals were obtained for the *artA-* and the *artB, C* reporter. When trap formation was induced after addition of *C. elegans*, fluorescence for *artA-C* was not detected in newly formed but in older traps and in conidia and chlamydospores (Fig. 2b, Supplementary Fig. S9). These results suggest different spatially and timely regulated promoter activities for *artA* and *artB, C*.

To confirm the observed expression pattern obtained with the promoter-reporter construct and analyze if protein expression follows the same pattern, we tagged ArtA C-terminally with GFP and ArtB with mCherry *in locus* in the same strain. The constructs were integrated into the *D. flagrans* genome by homologous recombination and fluorescence detected in young hyphal tips, older substrate-, and aerial hyphae. The fluorescent signal of ArtA was strong in hyphal tips, whereas the signal for ArtB was almost undetectable (Fig. 2c). Older hyphae, spores, and older traps were highly fluorescent for both ArtA and ArtB (Fig. 2c).

**Fig. 3:**
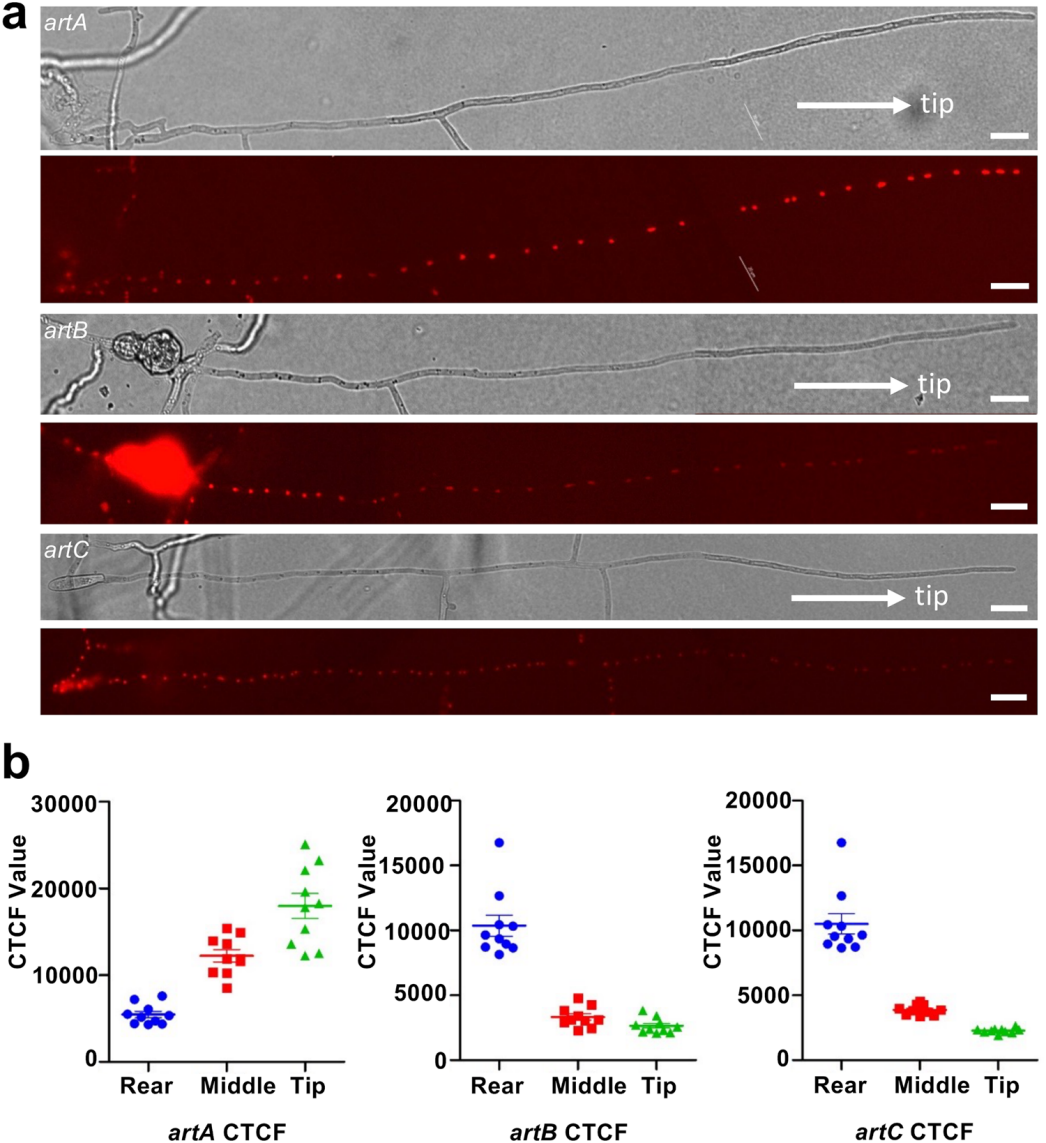
Monitoring the promoter activity of *artA-C* in a *D. flagrans* hypha. **a** Visualization of expression gradients of *art A* (strain SXY17)*, artB* (strain SXY20), and *artC* (strain SXY19) along a hypha. Scale bar, 20 µm. **b** Quantification of CTCF intensity values of nuclei in hyphae displayed in **a**. (Mean ± SD; *n* = 10 biologically independent samples.) Source data are provided as a Source Data file.

When observing longer hyphae of the above transcription-reporter strains, we noticed interesting expression gradients. Whereas *artA* expression decreased from the tip to the rear of the hyphae, the fluorescent signals of the *artB* and *artC* constructs were very weak in hyphal tips and young traps and appeared very strong in old hyphal parts and spores (Fig. 3). These results indicate strict spatiotemporal control of different genes of the *artA*-gene cluster and hence probably the production of 6-MSA in hyphal tips and the production of arthrosporols in older hyphae and older traps. Our results suggest that the *artA*-gene cluster is responsible for the production of pigments, arthrosporols, and 6-MSA. To our knowledge, this is the first example that the biosynthetic capacity of a secondary metabolite gene cluster is increased by temporal and spatial regulation of certain cluster genes.

### *C. elegans* ascarosides regulate arthrosporol and 6-MSA biosynthesis

**Fig. 4:**
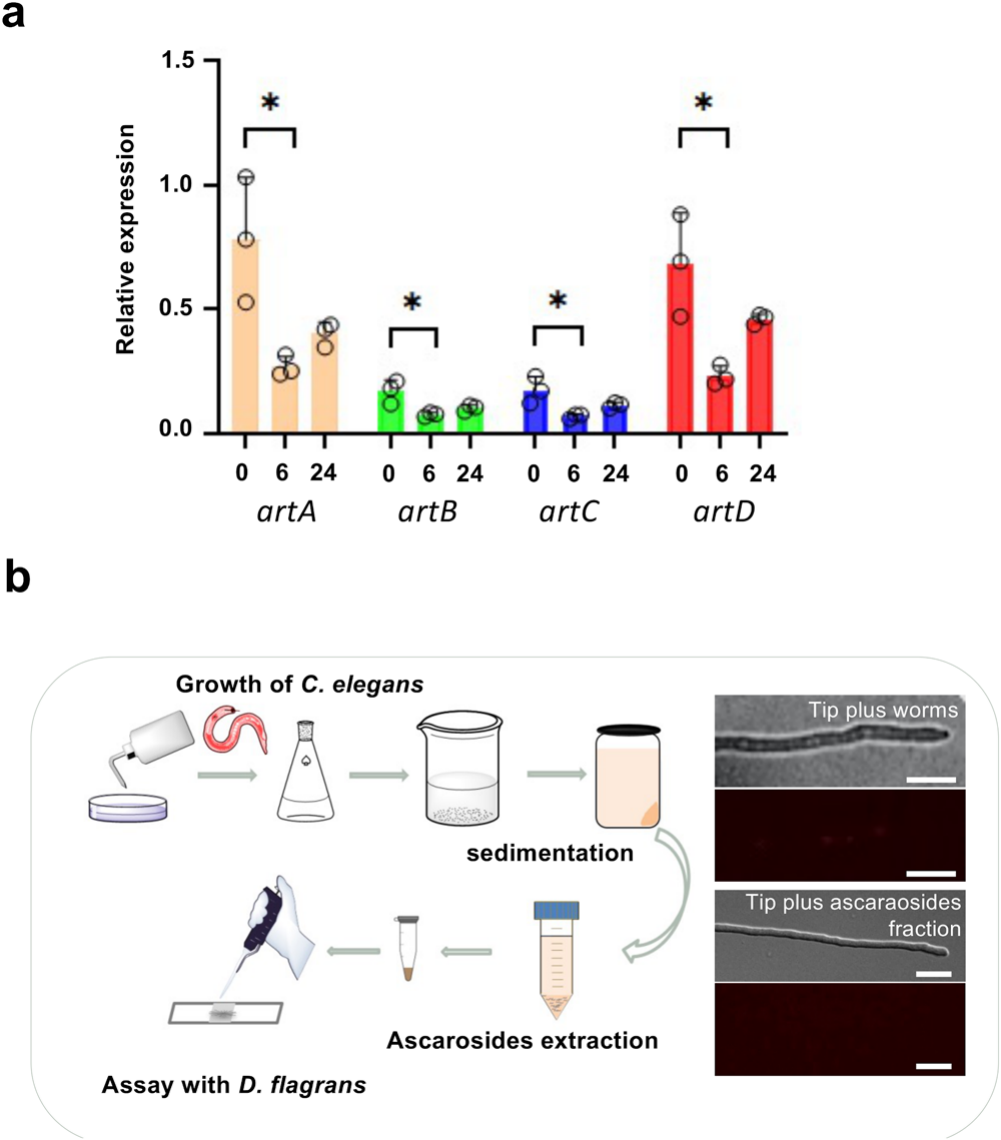
Regulation of *artA*-gene cluster gene expression by *C. elegans*. **a** Expression levels of four genes of the *artA* cluster *(artA*, *artB*, *artC*, and *artD*) were repressed after addition of *C. elegans*. *D. flagrans* spores were germinated on cellophane paper on LNA medium and grown overnight at 28 °C. Then seven-day-old cultured nematodes (about 50,000) were added and mycelia harvested at the times indicated and processed for quantitative real-time RT PCR analysis (mean ± SD, *n* = 3 biological with 3 technical replicates; asterisks represent significance levels as determined with the unpaired *t* test compared to WT; *p < 0.05, *artA* 0 h vs 6 h, *p*-value = 0.025; *artB* 0 h vs 6 h, *p*-value = 0.027; *artC* 0 h vs 6 h, *p*-value = 0.030; *artD* 0 h vs 6 h, *p*-value = 0.020). **b** Scheme of the ascaroside blend isolation (left, pictures were used from the ChemDraw software package). Microscopic visualization of the expression of *artA* (strain SXY17) after treatment with worms or the ascarosides blend in hyphal tips (right). Scale bar, 20 µm. Source data are provided as a Source Data file.

We showed that the *D. flagrans* mycelium produces arthrosporols and 6-MSA and both compounds inhibit trap formation. This raises the question how trap formation is induced in the presence of nematodes. To test the hypothesis that the presence of *C. elegans* causes repression of the production of the inhibitors arthrosporol and 6-MSA, we added *C. elegans* to growing mycelium of *D. flagrans* on low nutrient medium and studied the expression of *artA-D* by quantitative real-time PCR before adding the worms and 6 and 24 h after addition of the nematodes (Fig. 4a). In all four cases (*artA-D*), transcript abundances were reduced after 6 h and recovered slightly after 24 h. The expression levels varied to some extent between biological replicates. This may be due to variations of the number of traps, and the fact that the mycelium does not express the genes homogenously in all hyphae (see above). Therefore, we analyzed gene expression also at the cellular level using the established reporter construct for *artA* (Fig. 4b). Hyphal tips indeed did not show fluorescence in the presence of worms. The next question was how the presence of worms may affect the *artA* expression level. Because *C. elegans* ascarosides have been shown to induce trap formation in *A. oligospora*, we anticipated that these molecules could directly act on *artA-*gene expression^10^. As a first step to confirm this hypothesis, we applied the supernatant of a water solution washed off from culture plates of *C. elegans* (= worm water) to hyphae and observed the same downregulation of *artA*. Next, we followed a protocol for ascaroside extraction and could also show the effect with the crude ascaroside fraction (Fig. 4b).

### 6-MSA as chemoattractant for *C. elegans*

**Fig. 5:**
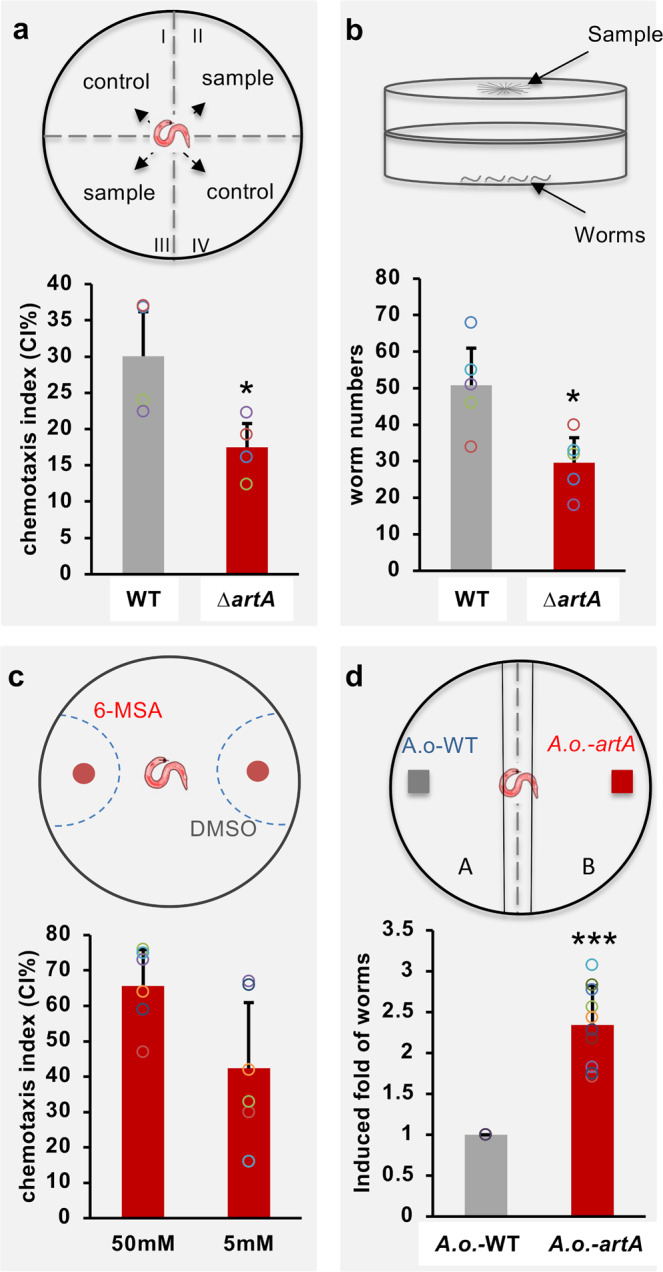
*C. elegans* is attracted by 6-MSA. **a** Four-point chemotaxis assay with direct physical contact of mycelia and worms. The chemotaxis index was calculated from 4 to 10 experiments. Three biological replicates. **b** Headspace chemotaxis assay. The fungal strains were grown in the lid of a petri dish and the nematodes in the bottom. **c** Chemotaxis assay with 6-MSA on agar plates. Nematodes were initially placed in the center of the plate. In all three assays, a score of 100% represents maximum attraction. Assays for each condition were repeated at least six times on at least two different days. **d** Chemotaxis assay with *A. oryzae* (transformed with empty vector) or *A. oryzae* expressing *artA*. Nematodes were initially placed in the center of the plate. *Y*-axis represents the induced fold of worms’ number, compared with the *A. oryzae* control (set as a score of 1, *n* = 10). Data were expressed as mean ± SD. The error bar represents the standard deviation. Asterisks represent significance levels as determined with the unpaired *t*-test compared to WT; *p < 0.05, **p < 0.01, ***p < 0.001. **a** *p*-value = 0.031290533; **b** *p*-value = 0.013388722; **d** *p*-value = 5.57371E-08. Source data are provided as a Source Data file.

The spatial expression patterns of *artA-C* suggested the production of 6-MSA at hyphal tips and the production of arthrosporols further back and in older hyphae and traps. Both compounds inhibit trap formation, and 6-MSA also inhibited germination and hyphal extension. However, the question was if 6-MSA could play an additional role at hyphal tips. Another salicylic acid derivative, its methyl ester (MSA), also known as wintergreen oil, is a well-known signaling compound in plant–pathogen interactions^27,28^. Plants under herbivore attack may use MSA to attract enemies of the herbivores^29^. It was also shown that such volatiles may indicate putative host plant roots for herbivores like root-knot nematodes^30^. Therefore, we tested if 6-MSA could be involved in the fungal–nematode interaction (Fig. 5). We first tested the WT and the ∆*artA-*deletion strain in a chemotaxis assay. Wild-type colonies clearly attracted *C. elegans* independent if the mycelium was growing on the same agar medium as the nematodes or if the mycelium was placed in the center of a lid of a petri dish and the nematodes on the bottom (Fig. 5a, b). These results showed that *D. flagrans* produces volatile compounds to attract *C. elegans*. In the *artA-*deletion strain, the effect was reduced, suggesting that 6-MSA could be a chemoattractant. The remaining response was probably due to other compounds such as MMB (methyl-3-methyl-2 butenoate)^9^. To further test this hypothesis, we applied 6-MSA directly in the chemotaxis assay and found that it is active as chemoattractant (Fig. 5c). However, rather high concentrations were required for the effect, which may be due to the fact, that the chemical was mixed in a liquid agar sample and then applied to a side of the agar plate. To simulate a more natural situation, we tested the *A. oryzae* strain producing 6-MSA in the chemotaxis assay. Whereas the *A. oryzae* strain containing an empty vector, was not attractive for *C. elegans*, expression of *artA* made the strain attractive (Fig. 5d).

## Discussion

**Fig. 6:**
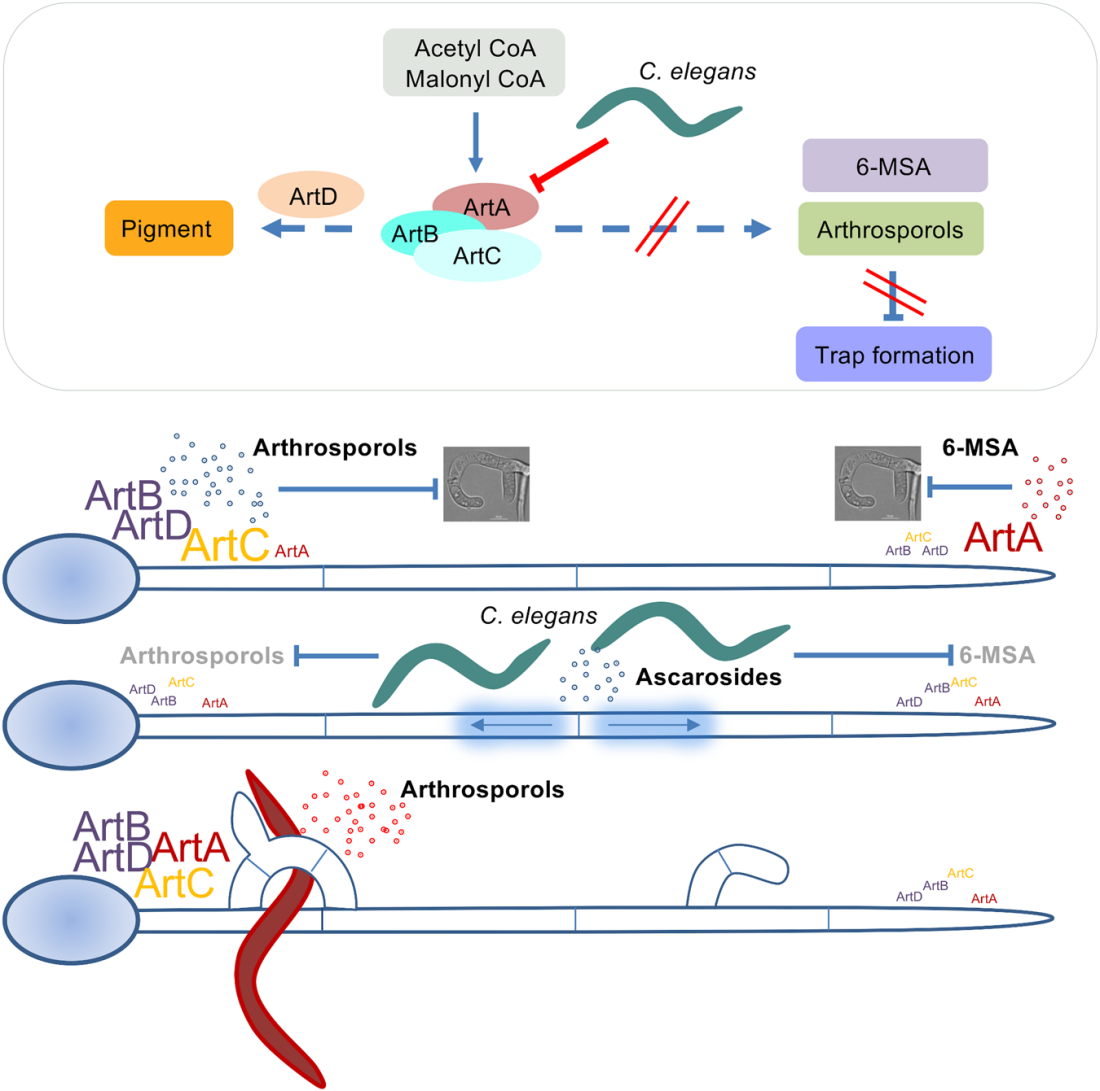
Scheme of the action of the *artA-*gene cluster and the effect of the presence of *C. elegans*. Gradients of 6-MSA, arthrosporols, and ascarosides control trap formation in *D. flagrans*. 6-MSA is produced at hyphal tips, while further back 6-MSA is converted to arthrosporols. Both compounds block trap formation. After successful attraction of a large number of nematodes by 6-MSA and other molecules, the concentration of ascarosides raises above a threshold. This will result in the downregulation of the *artA*-gene cluster genes, which in turn causes a decrease of 6-MSA and arthrosporols. If their concentration falls below a threshold, repression of trap formation is released, and traps are formed to catch the worms. After the digestion of trapped worms (red color), *artA*-gene cluster gene expression resumes, and increasing arthrosporols concentrations prevent unnecessary and excessive trap formation. Bigger font size symbolizes higher expression levels.

The interaction between nematodes and nematode-trapping fungi requires sophisticated inter-organismic communication with low-molecular weight signaling molecules. *C. elegans* produces a blend of different ascarosides, normally used for male attraction and other behavioral controls as well as developmental decisions^18^. On the other hand, the fungal counterpart produces various volatile compounds to lure nematodes into the traps^9^. In addition, the fungi produce a number of different sequiterpenyl expoxy-cyclohexenoids (SEC), which control trap formation. At least three arthrosporols, oligosporons, and arthrobotrisin were identified in extracts of *A. oligospora*^20,23^. The biosynthesis of these SECs has been studied with targeted gene-deletions followed by metabolome analyses^21,22,31^. A polyketide synthase is one of the central enzymes. Deletion of this polyketide synthase gene caused an increase of the number of traps, but it remained open if all or only some SECs are involved in the repression of trap formation and if certain intermediates would be important as well^21^. Here, we showed that the expression of individual genes of the *artA*-gene cluster are individually regulated in time and space and through the presence of nematodes, resulting in different cocktails of low-molecular weight compounds with different effects on hyphal development in different parts of the mycelium and dependent on the number of worms. In a colony without any worms the polyketide synthase ArtA is mainly produced at hyphal tips (Fig. 6). Because *artB-D* are not expressed in hyphal tips, 6-MSA should accumulate there. This compound prevents trap formation at the tip of the hypha. In the rear of the hypha, *artA* is expressed along with *artB* and *C*, leading to the production of arthrosporols, which also inhibit trap formation. If nematodes are added to the mycelium, *artA* is downregulated, thereby inducing trap formation. Young traps also do not express *artA* and therefore, new traps can be added resulting in trapping networks. However, old traps resume *artA-C* gene cluster expression and limit further trap development. If both, arthrosporols and 6-MSA inhibit trap formation, the question arises why the two compounds are required and why they are produced in different parts of the mycelium. One explanation could be the fact that 6-MSA has nematicidal activity^26^. This means that the hyphal tips, which are at the forefront of the growing mycelium and are the first ones with contact to nematodes, can act on nematodes and perhaps facilitate the trapping process. On the other hand, we found that 6-MSA attracts *C. elegans* and lures the worms into the mycelium.

The control of trap formation in *D. flagrans* is rather complicated and requires the integration of internal signals, such as starvation, with the presence of nematodes. It appears that fungal and nematode-derived low-molecular weight compounds are important for the regulation and the question arises why such a complicated system evolved. Diffusible molecules such as arthrosporols and 6-MSA can be produced within a colony and that way block trap formation in the entire mycelium. If now only one nematode moves across a colony, the nematode-derived ascarosides will downregulate the *art* genes in the hyphae in the vicinity of the worm. If this would lead to the induction of traps, several hours later mature traps would be ready, but then of course the nematode would be gone. Trap formation only makes sense in the presence of many nematodes. High numbers of nematodes will lead to high concentrations of ascarosides, which in turn will downregulate arthrosporol and 6-MSA formation in the entire colony. Hence traps will be formed in the entire colony. Therefore, arthrosporols/6-MSA and ascarosides can also be considered as quorum-sensing molecules.

Our work emphasizes the importance of methylated isoforms of salicylic acid as signaling molecules. Whereas the unmethylated form is used as plant hormone, the methylated volatile forms are important for long-distance communication^27,29,32^. It is noteworthy that the chemical origin of the molecules is very different. Whereas plants produce SA and MSA (the methyl ester) from cinnamic acid, a product of the shikimate pathway, fungal 6-MSA is the result of a polyketide synthase^33^. Hence, the two isoforms are examples for molecules with similar signaling functions but very different evolutionary origins.

## Methods

### Strains and culture conditions

*D. flagrans* (CBS 349.94) was obtained from the CBS-KNAW culture collection (The Netherlands). *D. flagrans* and *C. elegans* were cultured as described^15^ (http://www.wormbook.org). Unless otherwise specified, PDA (potato-dextrose-agar, Sigma) and PDB (potato-dextrose broth) were used as media for routine growth of the fungus. The liquid culture was shaken at 180 rpm, 28°C under standard indoor light conditions. A modified low nutrient-agar (LNA)^1^ (KCl 1 g/l, MgSO_4_ - 7H_2_O 0.2 g, MnSO_4_ - 4H_2_O 0.4 mg, ZnSO_4_ - 7H_2_O 0.88 mg, FeCl_3_ - 6H_2_O, 3 mg, Agar 10 g, pH 5.5) was used for the induction of trap formation and microscopy. All strains are listed in Table S1, oligonucleotides in Table S2, and plasmids in Table S3.

### RNA isolation and quantitative real-time PCR

**Method 1 without worms:** The same amounts of spores of different fungal strains were inoculated in liquid PDB medium in 3.5 cm petri dishes and cultivated for 2, 3, 4, and 6 days. The mycelium was harvested directly and frozen immediately in liquid nitrogen. **Method 2 with worms:** 10^6^ spores of wild type were inoculated on cellophane on the surface of LNA media and cultivated at 28 °C overnight. On the second day 7-day-cultured nematodes were washed off with DEPC water and added to the fungus. After 0, 6, and 24 h the induced mycelium was harvested from the cellophane paper and frozen immediately in liquid nitrogen. Total RNA was extracted with *E.Z.N.A*.^®^ Tissue DNA Kit from Omega (Norcross, Georgia). DNase digestion was performed using the TURBO DNA-free kit (Thermo Fisher; Waltham, Massachusetts) and the RNA was diluted to 50 ng/μl with DEPC water. Quantitative real-time PCR was performed with SensiFAST SYBR and a Fluorescein One-Step Kit from Bioline (Lueckenwalde, Germany) on a CFX Connect Real-Time PCR Detection System (Bio-Rad, Munich, Germany). Each reaction had a volume of 25 μl with 0.2 μM primers and 100 ng RNA according to the protocol of the kit. The program and melting curve analyses were carried out as described^15,34^. Fold changes were calculated using the formula 2^−(ΔΔCt)^. The gamma actin orthologue DFL_002353 was used as an internal reference gene for normalization. The qRT-PCR was performed using three biological replicates. Oligonucleotides used for real-time PCR are listed in Table S3.

### Plasmid construction

To create a C-terminal mCherry fusion of histone H2B under the control of different promoters, the related fragment including the promoter sequences were amplified by PCR, using *D. flagrans* genomic DNA as template (*artA* promoter: 1407 bp, amplified with primers artA(p)-h2b-fw and artA (p)-h2b-rev; *artB* promoter: 1141 bp, amplified with primers artB(p)-h2b-fw and artB(p)-h2b-rev; *artC* promoter: 1567 bp, amplified with primers artC(p)-h2b-fw and artC(p)-h2b-rev). The backbone of the plasmid containing the mCherry was amplified and assembled with the promoter fragment using the NEBuilder HiFi DNA Assembly Cloning Kit (New England Biolabs, Frankfurt). Q5 High-Fidelity DNA polymerase for PCR and restriction enzymes were purchased from New England Biolabs (Frankfurt, Germany).

The *artA*-cluster genes were deleted by homologous recombination. Around 1 kb flanks homologous to the 5′ and 3′ regions of the targeted gene were amplified by PCR, using *D. flagrans* genomic DNA as template (*artA*: 856 bp/1039 bp (amplified with primers artA olup_fw and artA olup_rev, artA oldown_fw and artA oldown_rev); *artB*: 1430 bp/931 bp (amplified with primers artBolup_fw and artBolup_rev, artBoldown_fw and artBoldown_rev); *artC*: 1141 bp/1067 bp (amplified with primers artColup_fw and artColup_rev, artColdown_fw and artColdown_rev);; *artD*: 1311 bp/912 bp (amplified with primers artDolup_fw and artDolup_rev, artDoldown_fw and artDoldown_rev)). The hygromycin-B resistance cassette hph was amplified using pFC332 as the template (1835 bp, (amplified with primers hygolup_fw and hygolup_rev)). All fragments for each gene were assembled into the pJET1.2 vector (Thermo Fisher, digested with *Eco*RV) using the NEBuilder HiFi DNA Assembly Cloning Kit (New England Biolabs, Frankfurt). Phusion polymerase (NEB) was used for PCR and the fragments contained 25 bp overlapping regions to the neighboring fragment. Standard transformation procedure and plasmid isolation for *Escherichia coli* were used.

### ArtA and ArtB protein co-localization

The *artA* and the *artB* gene were tagged by homologous recombination in the same strain and thereby integrated *in locus*. About 1 kb flanks homologous to the end of each gene before the stop codon and the 3′ regions of the targeted gene were amplified by PCR, using *D. flagrans* genomic DNA as template (*artA*: 956 bp/798 bp (amplified with primers pksAend_pjet_ol_for and pksAend_gfp_ol_rev, pksARB_hph_ol_for and pksARB_pjet_ol_rev); *artB*: 946 bp/940 bp (amplified with primers ArtBend_pjet_ol_for and ArtBend_mcherry_ol_rev, ArtBRB_H_ol_for and ArtBRB_pjet_ol_rev)). The hygromycin-B resistance cassette *hph* was amplified using pFC332 as template (1835 bp, (amplified with primers H_Gfp_ol_for and H-pksARB-ol-rev for *artA*, H_mcherry_ol_for and H_artBRB_ol_rev for *artB*). The *GFP* and *mCherry* cassettes were amplified (780 bp/762 bp, (amplified with primers Gfp_pksAend_ol_for and Gfp_H_ol_rev, Mcherry_artBend_ol_for and Mcherry_H_ol_rev). All fragments for each gene were assembled into the pJET1.2 vector (Thermo Fisher, digested with *Eco*RV) using the NEBuilder HiFi DNA Assembly Cloning Kit (New England Biolabs, Frankfurt). Phusion polymerase (NEB) was used for PCR and the fragments contained 25 bp overlapping regions to the neighboring fragment. Standard transformation procedure and plasmid isolation for *E. coli* were used.

### Protoplast transformation of *D. flagrans*

Protoplast transformation and gene targeting in *D. flagrans* were done as described^15^. Briefly, after cultivated for 24–36 h at 28˚C, 180 rpm, the mycelium was harvested and washed with MN solution (0.3 mol/l MgSO_4_, 0.3 mol/l CaCl_2_), and suspended in 10 ml MN buffer containing 4 mg/ml kitalase (Fujifilm Wako Chemicals) and 20 mg/ml VinoTaste Pro (Novozymes), followed by incubation for 2 h at 30˚C, 70 rpm. Protoplasts were collected by filtering through 3-layers of sterile miracloth tissue and centrifuged for 15 min at 2.400 x *g*. Protoplasts were washed twice with KTC (1.2 mol/l KCl, 10 mmol/l Tris-HCl pH 7.5) solution and finally resuspended in 700 μl KTC. Protoplasts were quantified using light microscopy. Hundred microliter of protoplasts (5 × 10^6^) were mixed with 5–8 μg of DNA and incubated for 2 min on ice. One milliliter of PTC (50% polyethylene glycol 6000, 20 mM Tris−HCl at pH 7.5, and 50 mM CaCl_2_) was added and mixed gently. After incubation at room temperature for 20 min, 10 ml PDSSA (24 g/l potato dextrose broth, 0.6 mol/l sucrose, 0.3 g/l peptone, 0.3 g/l tryptone, 0.3 g/l yeast extract, 14 g/l agar) was added to the transformation mixture and then poured onto PDA plates supplemented with 100 μg/ml hygromycin-B. Transformed colonies were selected after incubation at 28 °C for 5−7 days. After single-spore isolation, correct transformants were identified by PCR and Southern blot analyses.

### Germination and hyphal growth experiments

To determine pigmentation and hyphal growth, the same amount of spores (1 × 10^4^) of different strains were inoculated at the center of PDA agar plates and incubated at 28˚C. Hyphal growth was calculated by kymograph analysis using the Fiji software (https://fiji.sc). Colonies were photographed every day. An AxioObserver Z1 inverted microscope using a 10x/0.30 N.A. objective (Zeiss) was used to check hyphal growth and spore production.

### Trap induction and microscopy

To quantify traps, fungal spores were harvested after 10 days cultivation on PDA agar plates. A total of 1 × 10^4^ spores were transferred to a thin LNA plate, and around 100 individuals of *C. elegans* were added. Co-incubation was carried out at room temperature in darkness for 24 h at 28˚C. Trap formation was observed and quantified under the microscope. Conventional fluorescence images were captured at room temperature using a Zeiss Plan-Apochromat 63x/1.4 Oil DIC, EC Plan-Neofluar 40x/0.75, EC Plan-Neofluar 20x/0.50, or EC Plan-Neofluar 10x/0.30 objective attached to a Zeiss AxioImager Z.1 and Axio-CamMR. CTCF (Corrected Total Cell Fluorescence) = Integrated Density − (Area of selected cell × Mean fluorescence of background readings). The cell fluorescence was measured using Image J. (https://theolb.readthedocs.io/en/latest/imaging/measuring-cell-fluorescence-using-imagej.html, contributed by Luke Hammond, the University of Queensland, Australia.)

Fluorescence recovery after photobleaching (FRAP) was used to check *artA*-mCherry expression of the hypha in *artA(p)::h2b::mCherry*. A total of 1 × 10^4^ spores were plated on thin LNA on coverslips, and *C. elegans* were added. After co-incubation for 12 h, a hypha was chosen under the microscope, bleached for 5 s and recovery monitored after 14 min (63x/1.4 Oil). FRAP was carried out using a Zeiss LSM 980, according to the Basic User Notes Airyscan.

### Inhibition of germination and trap formation by 6-MSA

6-MSA solution was prepared (1 mM) and diluted to different concentrations in DMSO. Solvent was used as control. Fungal spores were harvested after 10 days cultivation on PDA agar plates. A total of 1 × 10^6^ spores were transferred to a thin LNA agar containing different concentrations of 6-MSA (1, 2, 5, 10, and 20 mM) and the numbers of germinated and ungerminated spores were counted under the microscope. The experiment was repeated five times. To quantify the number of traps, 1 × 10^6^ spores were inoculated on a thin LNA plate, incubated for 1 day at 28˚C. On the second day, different amounts of 6-MSA were added to the medium, leading to final concentrations of 222, 250, 286, 333, 400, 500, and 667 µM. Then around 100 individuals of *C. elegans* were added. Co-incubation was carried out in the dark for 24 h at 28˚C. Traps were quantified under the microscope.

### Fermentation and extraction procedures

The same amounts of spores of *D. flagrans* strains were inoculated into 250 ml flasks containing 100 ml PDB medium and shaken at 180 rpm at 28°C for 8 days. The culture broths were filtered to separate the mycelia from the liquid. The mycelial pellets were extracted with 20 ml ethyl acetate and the supernatant extracted with ethyl acetate (1:1, v/v) for 1 h at room temperature. The organic fractions from the two parts were mixed and evaporated to dryness, then dissolved in 500 μl of methanol, filtered through 0.22 μm membranes, and further analyzed using high-performance liquid chromatography (HPLC−DAD) and gas chromatography−mass spectrometry (GC−MS) or HPLC–MS/MS.

### HPLC–DAD analyses

The samples were analyzed by HPLC (Agilent 1100, Waldbronn, Germany). The system was equipped with a Hypersil ODS RP C-18, 3 μm, 4 (i.d.) × 100 mm column (Thermo Fisher Scientific, Karlsruhe, Germany)^21^. The total flow rate was 1 ml/min with the mobile phase A with 0.1% formic acid in water and the mobile phase B with 0.1% formic acid in acetonitrile. The total running time was 30 min, starting at 10% of eluent A and 90% of eluent B for 2 min, followed by a linear gradient to 90% of eluent A over 18 min, holding for 4 min and back to the starting conditions to equilibrate the column for the next run. The column temperature was maintained at 40 °C. The injection volume for the extracts was 20 μl.

### HPLC–HRMS analyses

HPLC–HRMS analysis was carried out according to a modified method^35^. Mass spectrometric analyses were done using an X500R™ high-resolution ESI-Q-ToF mass spectrometer (Sciex, Toronto, Canada) equipped with an electrospray ionization (ESI) source operating in the positive ion mode as described before^25^. The HPLC–MS method was used to quantify 6-MSA (method 1). Arthrosporols were analyzed by their representative molecular ions or/and (?) quantitative ions. Automatically performed MS/MS experiments revealed the identity of the compound due to the specific fragmentation patterns (method 2)^20^. For the characterization of arthrosporols, the parameters were as follows: quantitative ions *m/z* [M-H]^-^/fragment ions: 393.1/138.9*, qualitative ions *m/z*: 121.2/373.2, declustering potential *U/V*: -29/-29/-29, collision energy *U/ev*: -13/-28/-13. Nitrogen was used as nebulizer, curtain and collision gas in all the experiments. Data analyses were done using the Origin software.

### GC–MS analyses

GC–MS analysis was performed on a GC-2010 Plus system (Shimadzu Corp., Kyoto, Japan) equipped with an DB-5(MS) column (5%-phenyl/95%-methylpolysiloxane; 30 m length x 0.25 mm i.d. x 0.25 µm film thickness; Agilent Technologies, Santa Clara, CA, USA). The initial column temperature of 100 °C was ramped at 5 °C/min up to 300 °C. Helium at a flow rate of 40 cm/s was used as carrier gas with Split injection mode (ratio 1:30). Extracts were dissolved in 1 ml methanol using a sonification bath for 20 min. Samples were centrifuged and the supernatant was used for GC–MS analysis. The injection temperature was 280 °C, the transfer line was held at 300 °C, and electron impact mass spectra were recorded at 70 eV. Peak identification was accomplished via the NIST11 Standards Reference Database integrated with the GC/MS solution software (version 2.72).

### Generation of “worm water”

A total of 10^6^ spores of *D. flagrans* were spread on LNA agar medium and grown for 24 h at 28^°^C. 7-day-cultured nematodes were washed off with 500 μl water and the suspension was kept at room temperature for 4 h. Then the suspension was centrifuged at 2,400 × *g* for 10 min and the supernatant (“worm water”) was added to germinated spores. After 24 h the expression levels of the proteins were checked in the microscope.

### Ascarosides isolation

For the isolation of a blend of ascarosides, we followed essentially a published protocol^36^. A total of 90,000 worms were inoculated into 150 ml culture medium and incubated at 20 °C for 9 days with shaking at 225 rpm. The culture was then kept on ice for 30 min to 1 h before the worms were sedimented by centrifugation (19,000 × *g*, 30 min). The supernatant was freeze-dried, and the dried material dissolved in 20 ml chloroform: methanol (3:1). After evaporation of the solvent, the residual compounds were dissolved in 1 ml ddH_2_O, centrifuged to remove unsoluble material, and the supernatant freeze-dried a second time. The final material was resuspended in 0.5 ml ddH_2_O and filtered through a membrane (22 µm pore diameter). The extracted ascaroside blend was added to the targeted samples on the microscopic slides.

### Chemotaxis assays

Chemotaxis assays were performed on 9 cm petri dishes containing LNA medium. **Method 1**: Direct contact bioassay: The same amount of spores were inoculated on LNA medium (1 × 10^6^ spores/μl) for 36 h, then about 300 nematodes were added to the center of the plates. To minimize the effects of adaptation, sodium azide (an anesthetic) was used to immobilize animals at the attractant and control areas. The attracted worms on four sections were counted after 4 h. The chemotaxis index was calculated as described (the number of animals on sample quadrants minus the number of animals on control quadrants)/(total number of animals)^9^. **Method** 2: Non-direct contact bioassay: Fungal strains and the nematodes shared the same atmosphere but had no direct physical contact. The two half petri dishes were sealed together with Parafilm and then incubated in a dark chamber at 28 °C. The bottom of the petri dish containing the fungal lawn was inverted over a second petri dish bottom of identical size containing 1 ml suspension of mixed stage nematodes (about 300 nematodes). The attracted worms on the upper petri dish were counted after 2 days. **Method 3**: Volatile compounds bioassay: Diluted 6-MSA (in DMSO) was mixed with agar and added into 1 cm diameter circles in the test area. The same volume of solvent, DMSO was mixed with agar and added in the control area. More than 50 animals (synchronized young adult animals were washed three times in S-basal buffer) were placed at the center and allowed to move freely for 1 h, then 5 μl of 1 M sodium azide was added to each side to immobilize the worms, before counting. The chemotaxis index is the number of worms on the test area minus the number of worms on the solvent area divided by the sum of worms from both areas. **Method 4:** About 1 cm^2^ of PDA medium with the fungal colony of *A. oryzae* (transformed with empty vector) or the *A. oryzae artA*-expressing strain was placed in the test or control location of the Petri dish, opposite from each other. More than 50 synchronized young adult nematodes were directly added in the center of the plate. After 1.5 h, 5 μl of 1 M sodium azide was added to each side to immobilize the worms. The plates were further incubated for 0.5 h before the attracted worms on each side were counted. The paired test was applied for the analysis.

### Statistics and reproducibility

Group sizes were based on previous experiments and are described in the figure legends or source materials. Unless specifically noted, each experiment was repeated three or more times independently. Data were collected from three biological and three technical replicates, unless otherwise noted. Data shown in column graphs or scatter plots represent mean ± the standard deviation (SD), as indicated in the figure legends. Plotted data points are shown. Statistical analysis was performed with ChemDraw Professional 15.1, GraphPad Prism 8.0.2, and OriginPro 2016. Details were given in the above methods and source data files.

### Reporting summary

Further information on research design is available in the Nature Research Reporting Summary linked to this article.

## Supplementary information

Supplementary Information

Reporting Summary

## Data Availability

All data and strains are available from the authors upon request. All data generated or analyzed during this study are included in this published article (and its Supplementary Information files or source data file). The *art* genes correspond to the following loci in the *Duddingtonia (Arthrobotrys) flagrans* genome (BioProject number PRJNA494930): DFL_002601 (*artA*), DFL_002602 (*artB*), DFL_002603 (*artC*), DFL_002604 (*artD*), DFL_002606 (*artE*), DFL_002600 (*artR*). Source data are provided with this paper.

## Source data

Source Data
