# Author Correction: Fatal attraction of *Caenorhabditis elegans* to predatory fungi through 6-methyl-salicylic acid

**DOI:** 10.1038/s41467-021-27538-4

**Published:** 2021-12-08

**Authors:** Xi Yu, Xiaodi Hu, Maria Pop, Nicole Wernet, Frank Kirschhöfer, Gerald Brenner-Weiß, Julia Keller, Mirko Bunzel, Reinhard Fischer

**Affiliations:** 1 Karlsruhe Institute of Technology (KIT) - South Campus, Institute of Applied Biosciences, Department of Microbiology, Fritz-Haber-Weg 4, Karlsruhe, Germany; 2 Karlsruhe Institute of Technology (KIT) - North Campus, Institute of Functional Interfaces, Department of Bioengineering and Biosystems, Eggenstein Leopoldshafen, Germany; 3 Karlsruhe Institute of Technology (KIT) - South Campus, Institute of Applied Biosciences, Department of Food Chemistry and Phytochemistry, Adenauerring 20 A, Karlsruhe, Germany; 4 Present Address: Shanghai Engineering Research Center of Hadal Science and Technology, College of Marine Sciences, Shanghai Ocean University, Shanghai, China

**Keywords:** Chemical ecology, Cellular microbiology, Fungal biology, Fungal ecology

Correction to: *Nature Communications* 10.1038/s41467-021-25535-1, published online 15 September 2021.

The original version of this Article contained an error in the Results section, which incorrectly read ‘The *artA-*deletion strain produced about 6000 times more 6-MSA than wild type.’ The correct version states ‘*artC*’ in place of ‘*artA*’. This has been corrected in both the PDF and HTML versions of the Article.

