# Supplementary material for: Fatal attraction of Caenorhabditis elegans to predatory fungi through 6-methyl-salicylic acid
Source: Nat Commun. 2021 Sep 15;12:5462. doi: 10.1038/s41467-021-25535-1 (PMC8443565; doi:10.1038/s41467-021-25535-1)
Supplement: Supplementary file 1 — Supplementary Information [file 41467_2021_25535_MOESM1_ESM.pdf]

Supplementary Information

Fatal attraction of *Caenorhabditis elegans* to predatory fungi through 6-methyl-salicylic acid

Xi Yu[1, 4], Xiaodi Hu[1], Maria Mirza[1], Nicole Wernet[1], Frank Kirschhöfer[2], Gerald Brenner-Weiß[2], Julia Keller[3], Mirko Bunzel[3] and Reinhard Fischer[1]*

running head: Methyl salicylate as morphogen and quorum sensing molecule in *D. flagrans*

Address: [1]Karlsruhe Institute of Technology (KIT) - South Campus
Institute of Applied Biosciences
Dept. of Microbiology
Fritz-Haber-Weg 4
D-76131 Karlsruhe, Germany
Phone: +49-721-6084-4630
E-mail: reinhard.fischer@KIT.edu
Homepage: www.iab.kit.edu

[2] Karlsruhe Institute of Technology (KIT) - North Campus
Institute of Functional Interfaces
Bioengineering and Biosystems
Hermann-von-Helmholtz-Platz 1
D-76344 Eggenstein Leopoldshafen

[3] Karlsruhe Institute of Technology (KIT) - South Campus
Institute of Applied Biosciences
Dept. of Food Chemistry and Phytochemistry
Adenauerring 20A
D-76131 Karlsruhe

[4] present address:
Shanghai Engineering Research Centre of Hadal Science and Technology, College of Marine Sciences, Shanghai Ocean University
Huchenghuan Road 999
201306, Shanghai, China

* corresponding author

Suppl. Figures

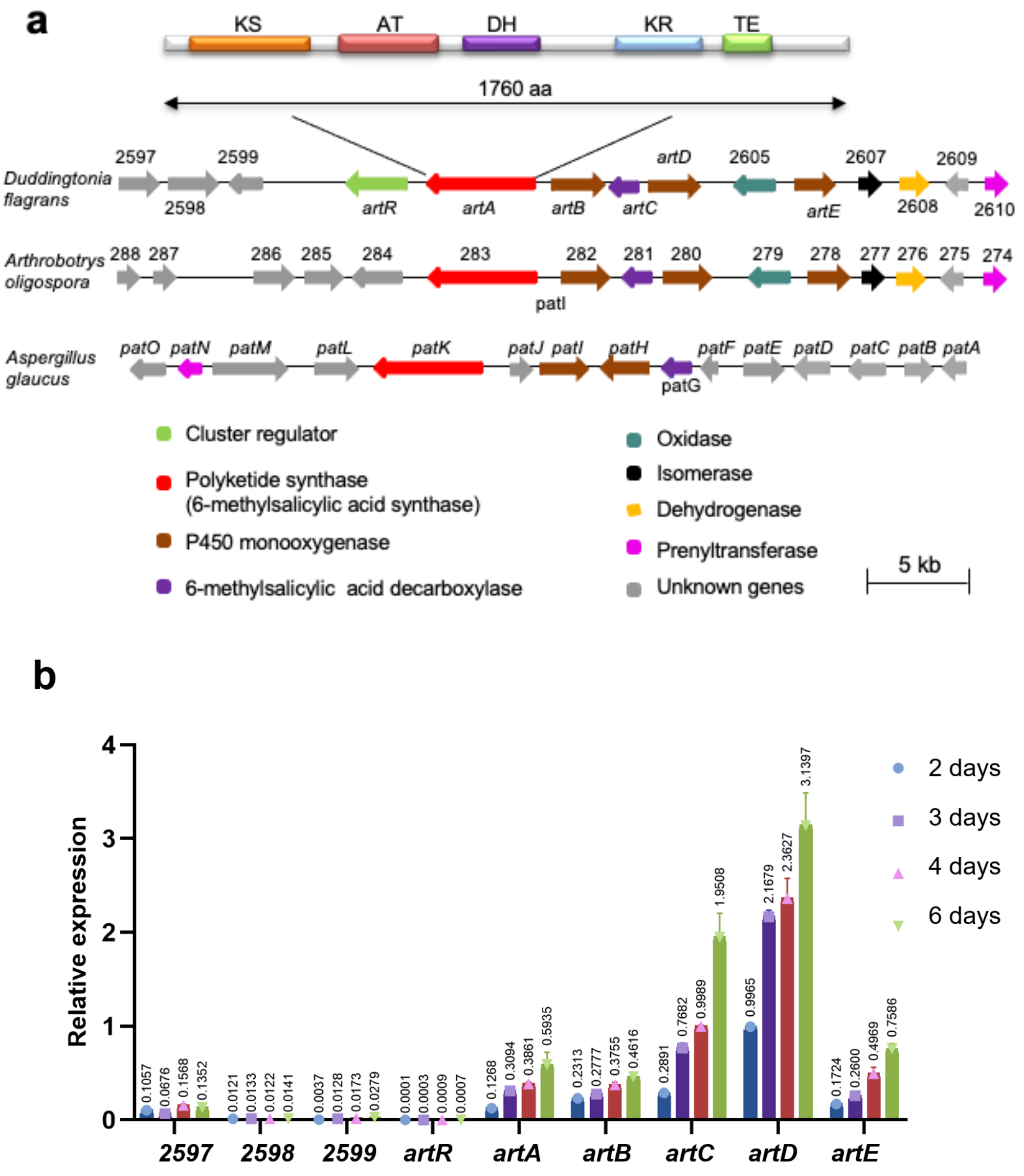

Suppl. Fig. S1: Characterization of the *artA*-gene cluster. (a) Bioinformatic analysis and comparison with the corresponding clusters in *Arthrospora oligospora* and the patulin gene cluster of *Aspergillus glaucus*. The *artA* gene (DFL_002601) is 5363 bp in length and encodes a 1760-amino acid long protein. It contains five typical catalytic domains, including a Keto Synthase (KS), an acyltransferase (AT), a dehydratase (DH), a β-ketoacylreductase domain (KR), and an acyl carrier protein (ACP) for the PKS. **(b)** Expression analysis of several genes indicated in **(a)**. RNA was extracted after two, three, four and six days and analyzed by quantitative real time RT PCR. Data are presented as mean values +/- SD, *n* = 3 biological with 3 technical replicates. Source data are provided as a Source Data file.

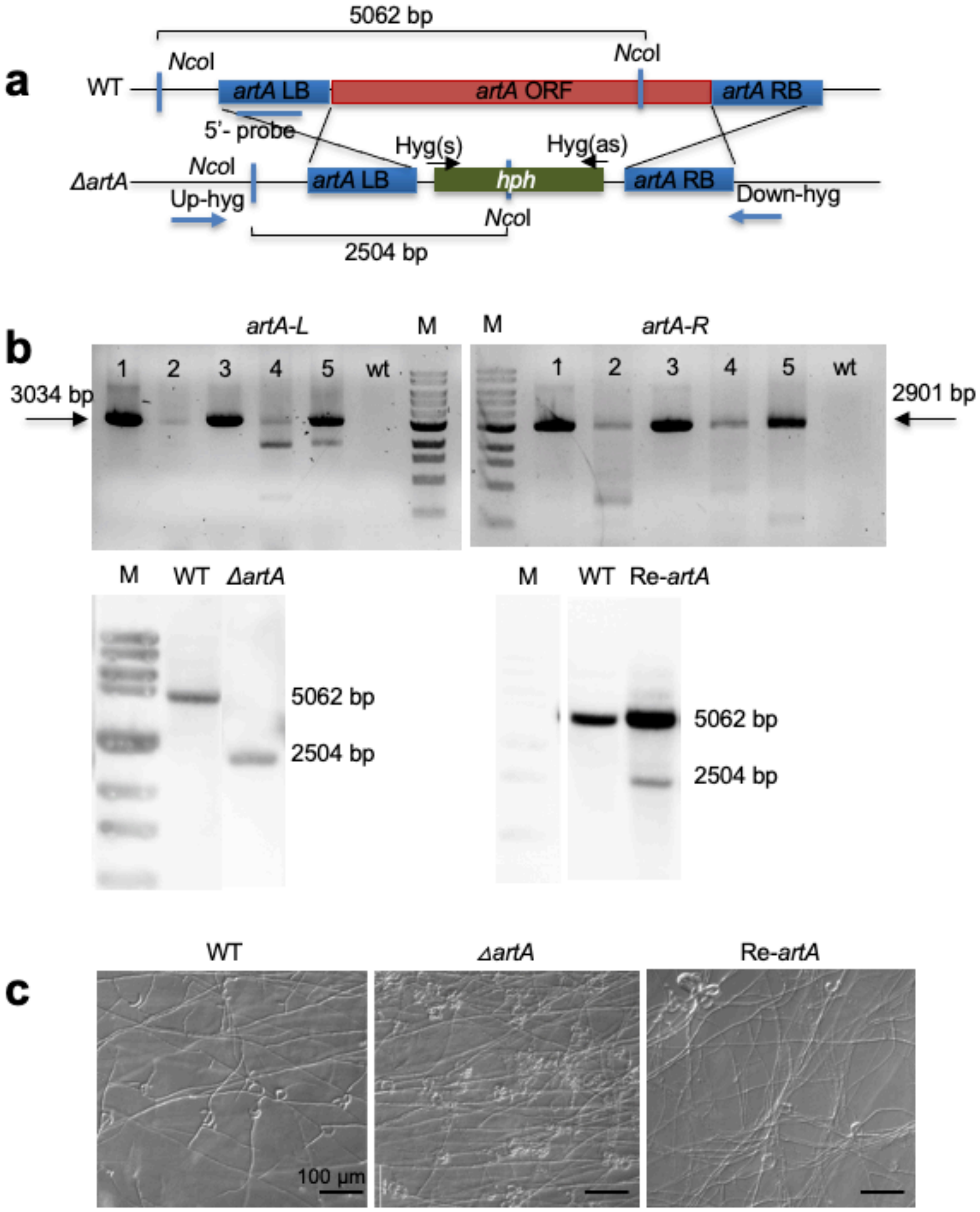

Suppl. Fig. S2: **(a)** Scheme of the deletion strategy for *artA*. **(b)** (upper panels) PCR analysis of WT and five independent transformants. The left part (*artA-L*) was amplified with the Up-hyg and Hyg(as), and the right part (*artA-R*) with Hyg(s) and Down-hyg. (lower panels) Southern blot analysis of the WT and Δ*artA*-deletion strain (SXY05) and a re-complemented strain (SXD05). Genomic DNA was digested and hybridized as indicated in **(a)**. **(c)** Overview of traps formed in colonies on agar plates (9 cm diameter) of the strains described in **(b).** Scale bar, 100 µm. Source data are provided as a Source Data file.

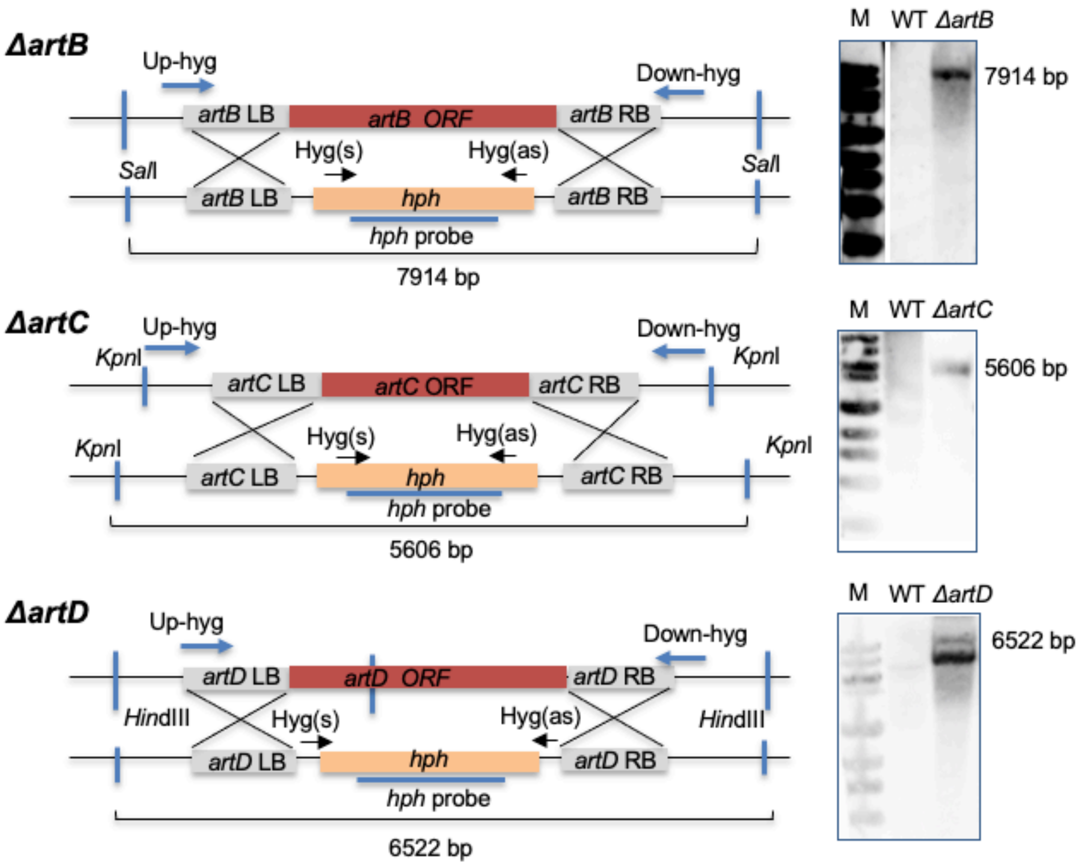

Suppl. Fig. S3: Schemes for the deletion of *artB-D* and results of the Southern hybridization using the *hph* gene as probe. Strains were SXY15 (Δ*artB*), SXY14 (Δ*artC*) and SXY16 (Δ*artD*). Source data are provided as a Source Data file.

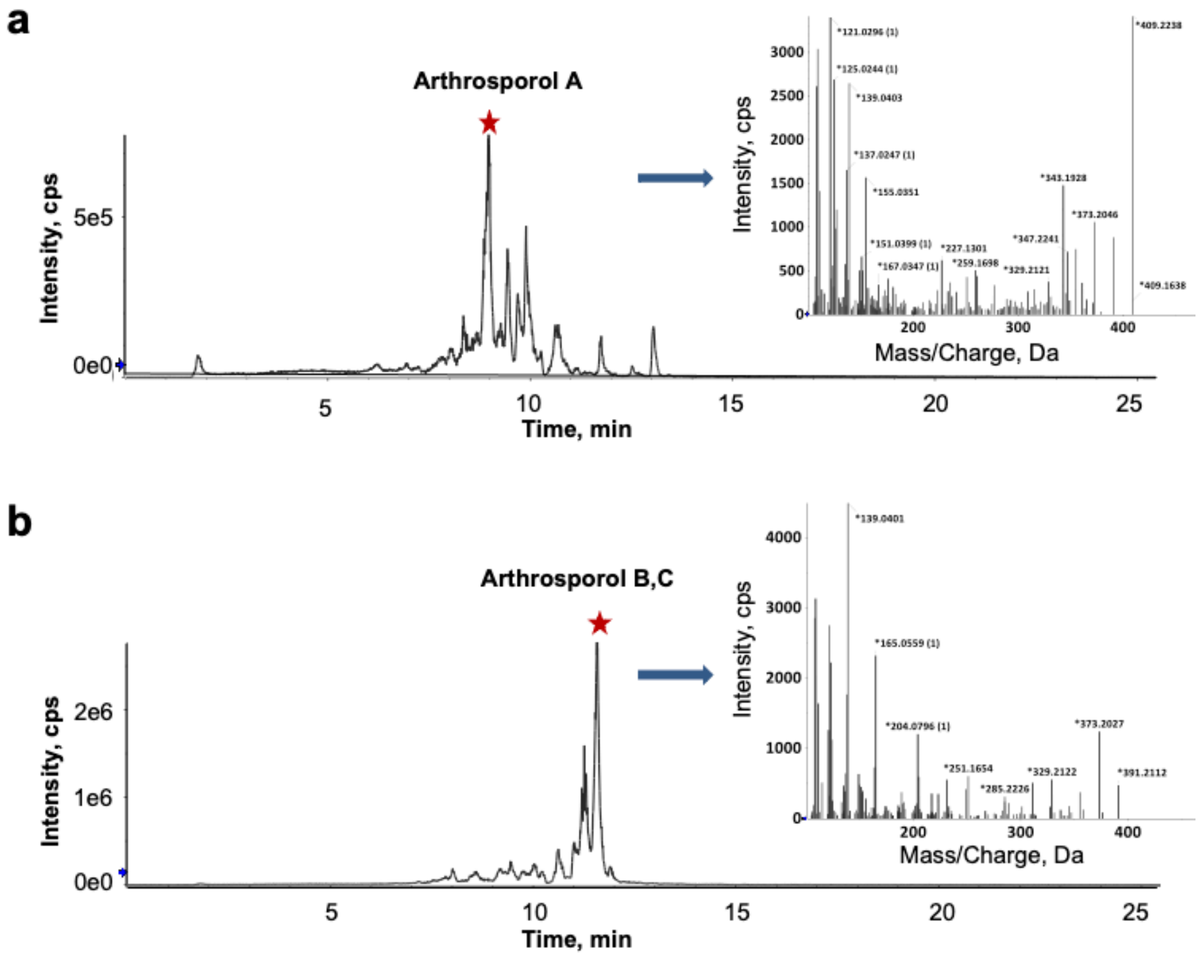

Suppl. Fig. S4: (a) LC-MS/MS characterization of arthrosporol A of the culture broths of wild type and the *ΔartA*-deletion strain. IDA TOF MS (100-1000) from 6.821 min, 409.223 +/-0.005 Da, noise filtered (noise multiplier=1.5), Gaussian smoothed (0.5 points). (**b)** Quantitative product ion; IDA TOF MSMS (70-1000) from 6.825 min. Precursor 409.2 Da, +1, noise filtered (noise multiplier=1.5), Gaussian smoothed (0.5 points). Quantitative and Qualitative ions m/z MS/MS of 409.223, 139.1, 155.0, 391.4. Source data are provided as a Source Data file.

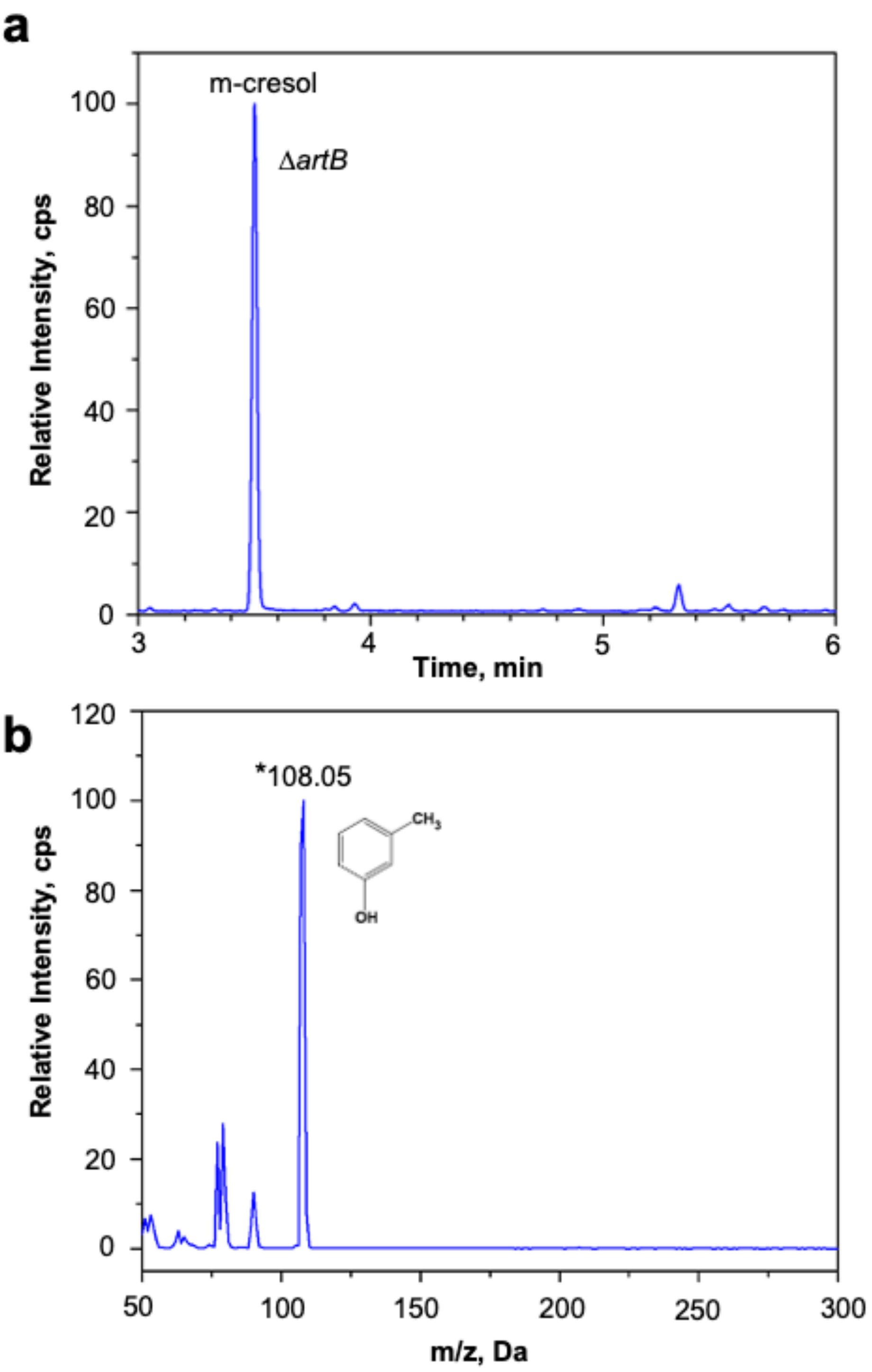

Suppl. Fig. S5: (a) GC-MS/MS characterization of m-cresol of the cultural broths of the *ΔartB*-deletion strain. Retention time of m-cresol: 3.5 min. **(b)** MS spectrum of the cultural broth of *ΔartB* for m-cresol: Raw spectrum, 3.470 -> 3.542 (scan: 283 -> 326); base peak, m/z 108.05 (Inten: 2,106,625). Source data are provided as a Source Data file.

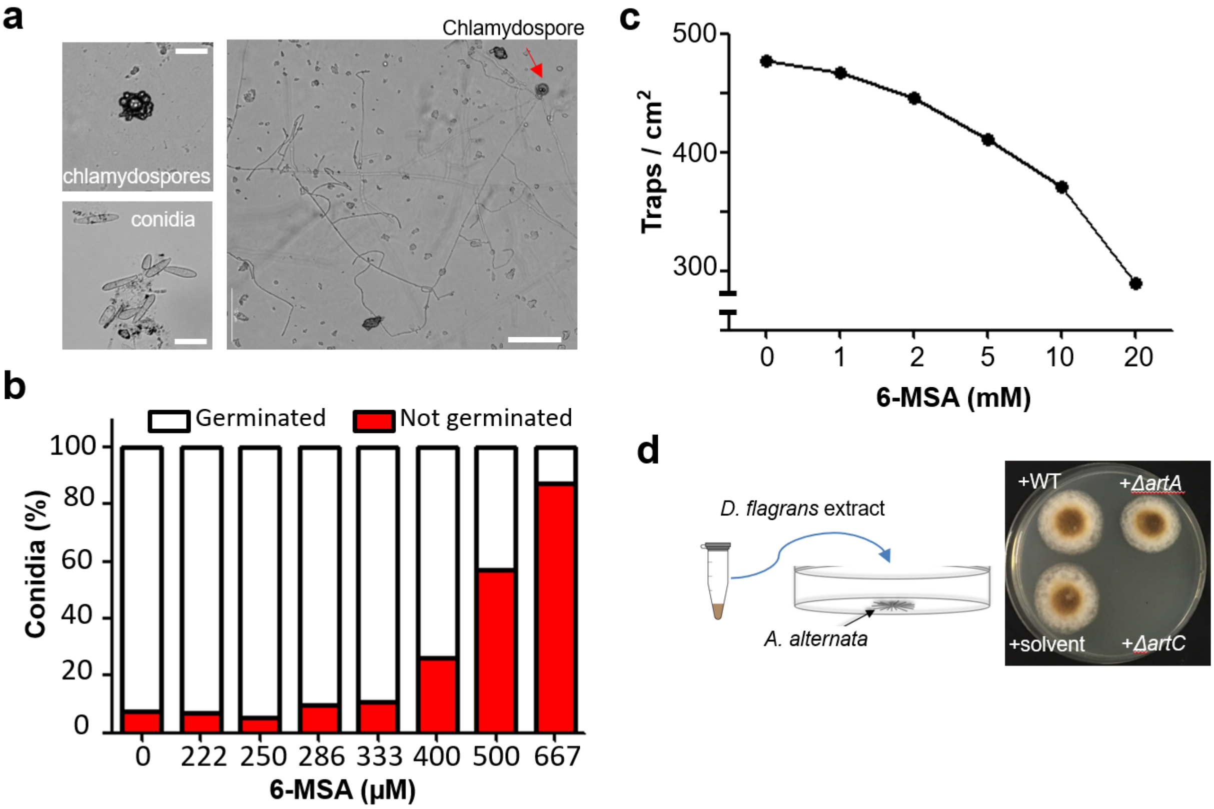

Suppl. Fig. S6: (a) Incubation of chlamydospores and conidia in the presence of 10 µl (undiluted)(left, Scale bar, 20 µm) and 10 µl (diluted 1:10 with the solvent) (right, Scale bar, 100 µm) extract. **(b)** Germination of spores was determined at different concentrations of 6-MSA. **(c)** Quantification of the number of traps a different 6-MSA concentrations. **(d)** Effect of *D. flagrans* extracts of cultural broth from WT and the Δ*artA* and Δ*artC* mutants on colony growth of *Alternaria alternata*. Source data are provided as a Source Data file.

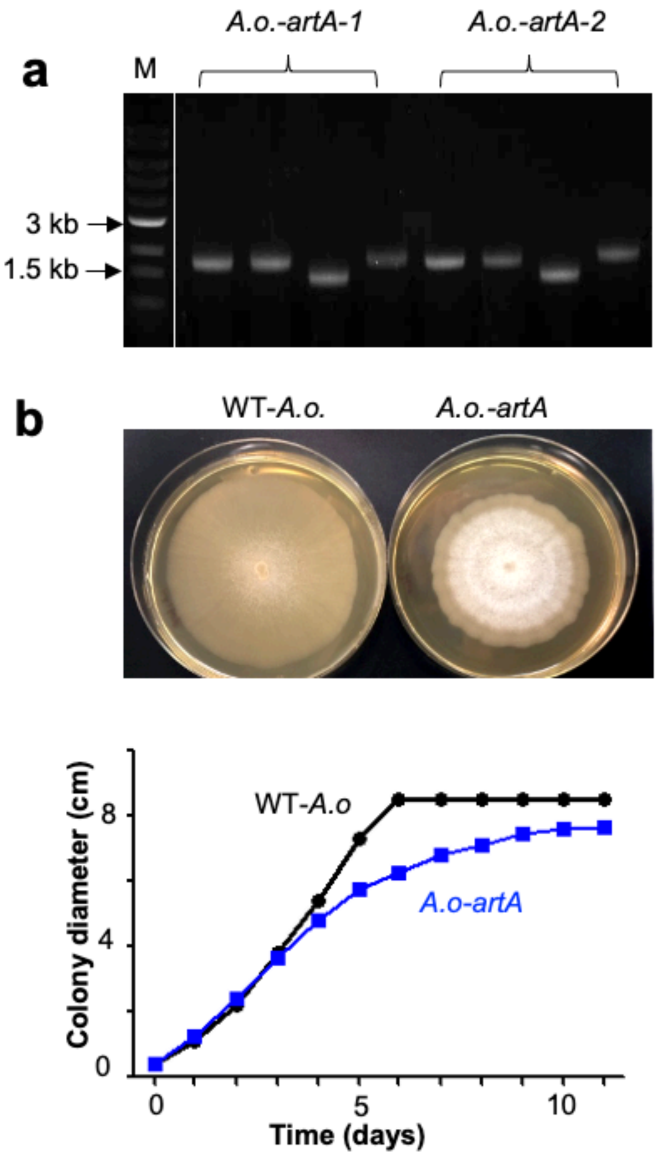

Suppl. Fig. S7: Heterologous expression of *artA* in *A. oryzae.* (a) (left) PCR analysis of different transformants. Left lane: Two transformants *artA-1* and *artA-2* and WT were chosen for further analysis. Four fragments were amplified with primers pksA_Ao_transform_for and pksA_AO_tf_up_rev (1639 bp), pksA_AO_tf_up_for and PksA _recomp_mid_rev (1623 bp), PksA _recomp_mid_for and pksA_AO_tf_down_rev (1343 bp), pksA_AO_tf_down_for and pksA_Ao_PCRconfirm_rev (1719 bp). (right) Two colonies of WT and the *artA*-expressing strain (SXD01) after 11 days of growth on PDA medium. **(b)** Quantification of fungal growth by measuring the colony diameter. Source data are provided as a Source Data file.

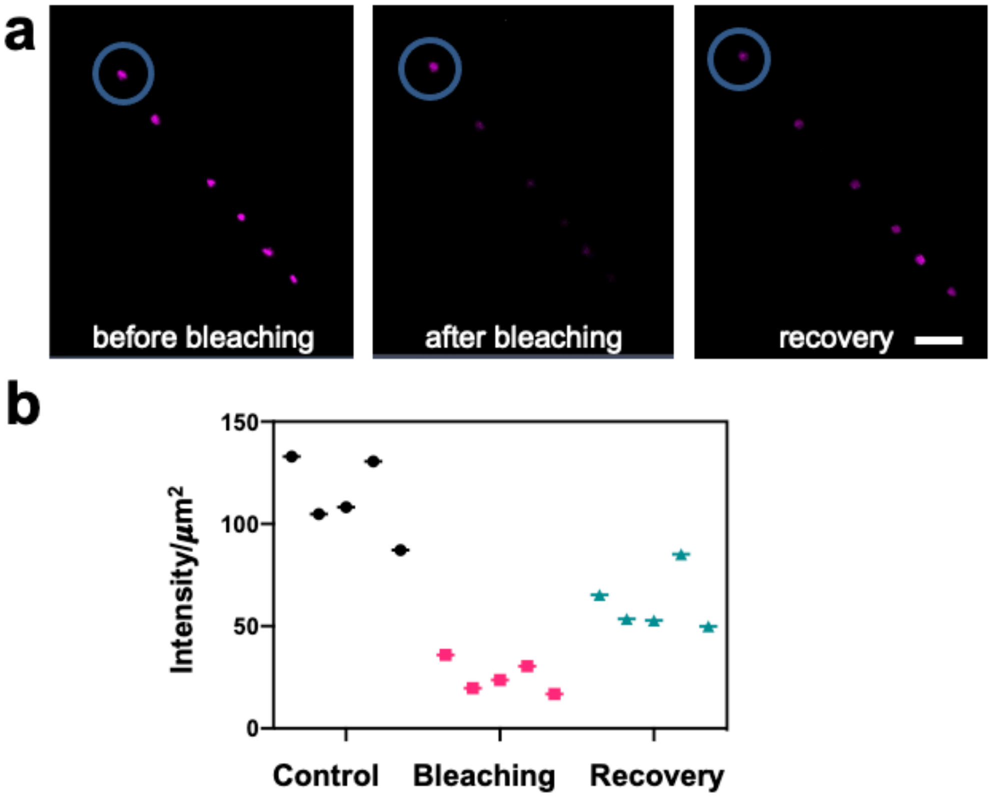

Suppl. Fig. S8: Promoter activity of *artA* in *D. flagrans*. The promoter of *artA* was fused to *h2b* and *mCherry* (strain SXY17). (**a)** Fluorescence recovery after photobleaching (FRAP). The sample was bleached for 5 seconds and recovered for 14 minutes. (Scale bar, 10 µm) **(b)** Quantification of CTCF intensity value of 5 different nuclei (left) after bleaching 5 s and recovery for 14 min. Scale bar, 20 µm. Source data are provided as a Source Data file.

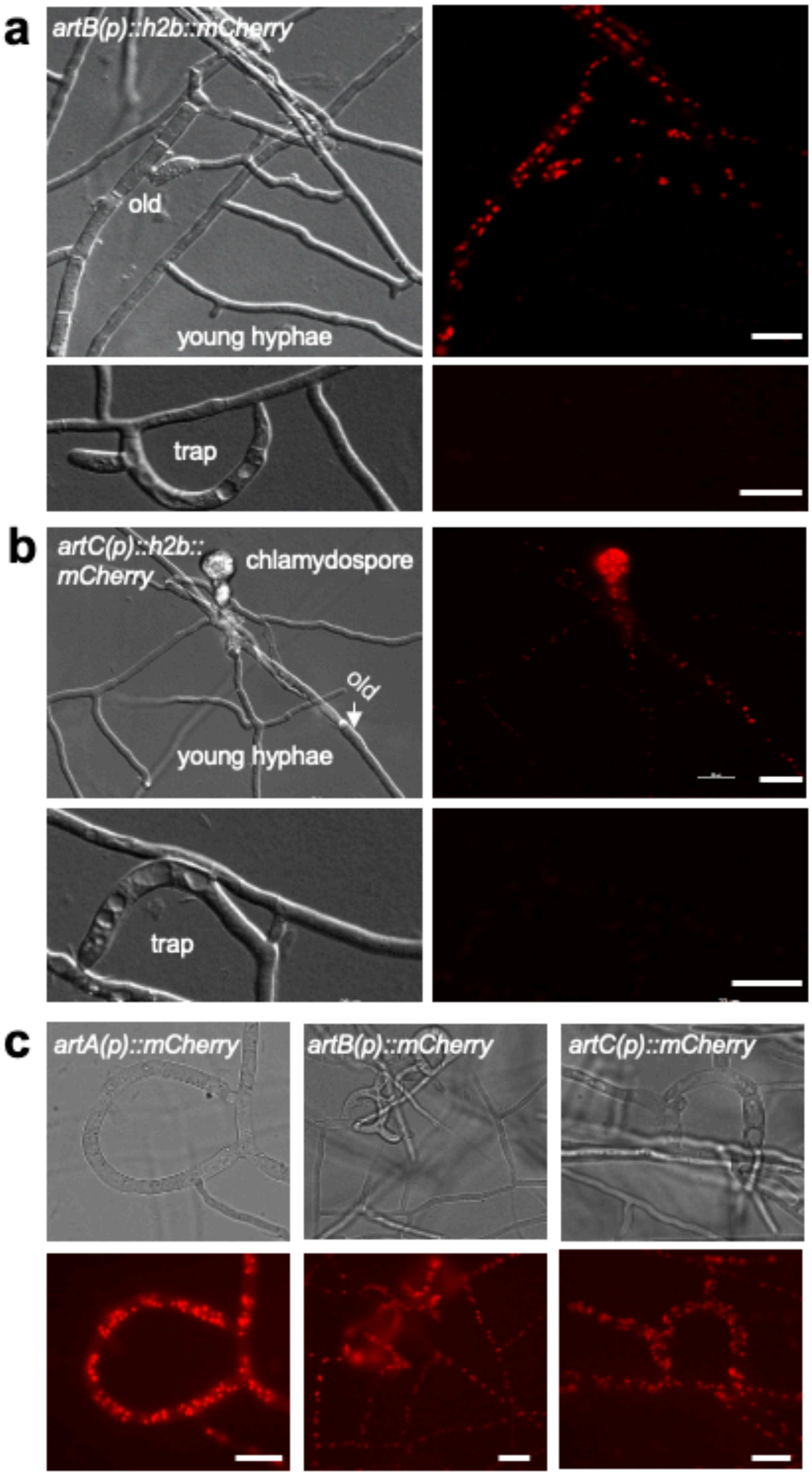

Suppl. Fig. S9: Expression analysis of *artA-C.* (a) Expression analysis of *artB* (strain SXY20). **(b)** Expression analysis of *artC* (strain SXY19). **(c)** Analysis of *artA-C* expression in old traps. Scale bar, 20 µm.

Suppl. Table S1: *D. flagrans* and *A. oryzae* strains used in this study.

Strain	Genotype	Reference
D. flagrans **CBS 349.94**	wild type (WT)	CBS-KNAW culture collection
**SXY05**	WT transformed with PXY09, Δ*artA::hyg*	This work
**SXY14**	WT transformed with PXY15, Δ*artC:*hyg,	This work
**SXY15**	WT transformed with PXY16, Δ*artB:*hyg	This work
**SXY16**	WT transformed with PXY17, Δ*artD:*hyg	This work
**SXY17**	WT transformed with PXY18, hyg, expression of *artA*	This work
**SXY20**	WT transformed with PXY21, hyg, expression of *artB*	This work
**SXY19**	WT transformed with PXY20, hyg, expression of *artC*	This work
**SXD05** ***Re-artA***	*artA* re-complementation; SXY05 transformed with pXD6	This work
**SXD53**	Co-localization of ArtA-GFP and ArtB-mCherry *in locus*, WT transformed with pXD88 and pXD89	
A. oryzae **Wild type**	Wild type	Russel J. Cox, Leibniz University
**SXD01** ***A.o-artA***	Heterologous expression of *artA* in WT, with pXD1	This work

Suppl. Table S2: Plasmids used in this study.

Name	Description	Reference
**pJET1.2**	ampR	Thermo Fisher
**pXY09**	*artA(l)::trpC(p)::hph::trpC(t):: artA(r); ampR;* plasmid for the deletion of *artA*	This work
**pXY15**	*artC(l)::trpC(p)::hph::trpC(t):: artC(r); ampR;* plasmid for the deletion of *artC*	This work
**pXY16**	*artB(l)::trpC(p)::hph::trpC(t):: artB(r); ampR* plasmid for the deletion of *artB*	This work
**pXY17**	*artD(l)::trpC(p)::hph::trpC(t):: artD(r); ampR* plasmid for the deletion of *artD*	This work
**pXY18**	*artA(p)::h2b::mCherry::TubT;* expression of *artA*	This work
**pXY21**	*artB(p)::h2b::mCherry::TubT* expression of *artB*	This work
**pXY20**	*artC(p)::h2b::mCherry::TubT* expression of *artC*	This work
**pXD6**	*pksA(p)::artA::artA(t); ampR; adeA* *artA* re-complementation	This work
**pXD1**	*amyB(p)::pksA::amyB(T); ampR; adeA* Heterologous expression of *artA* in *A. oryzae*	This work
**pNH30**	*gpdA(p)::mCherry::TubT*	[1]
**pXD88**	*artA::GFP::trpC(p)::hph::trpC(t)::artA(t)*	This work
**pXD89**	*artB::mCherry::trpC(p)::hph::trpC(t)::artB(t)*	This work

Suppl. Table S3: Oligonucleotides used in this study.

Name	Sequence (from 5' to 3')	Description
***artA* olup_fw**	CCGGATGGCTCGAGTTTTTCAGCAAGA TACATCCAATCCCCCCTAAC	*artA*-knockout-plasmid
***artA* olup_rev**	TCCACTAGCATTACACTTGGATCCcgAT CCCACCCATCCCAATTC	*artA*-knockout-plasmid
**hygolup_fw**	GGATCCAAGTGTAATGCTAGTGGA	*artA*-knockout-plasmid
**hygolup_rev**	CTGCAGTGGGGGGAGTTTAGGGAAA	*artA*-knockout-plasmid
***artA*oldown_fw**	TTTCCCTAAACTCCCCCCACTGCAGaa GCCAAAGAAGTCAAGTCAAAAG	*artA*-knockout-plasmid
***artA*oldown_rev**	TATTGTAGGAGATCTTCTAGAAAGATAC ATAACCGAACACCAAACAG	*artA*-knockout-plasmid
**artBolup_fw**	TGGCTCGAGTTTTTCAGCAAGATGTGG GCGAATAAGAATGAAAAG	*artB*-knockout-plasmid
**artBolup_rev**	TCCACTAGCATTACACTTGGATCCcgGA GCAAAGCGGAAACATAAAAG	*artB*-knockout-plasmid
**artBoldown_fw**	TTTCCCTAAACTCCCCCCACTGCAGaaA AGGAATACACGGGGCAAG	*artB*-knockout-plasmid
**artBoldown_rev**	TATTGTAGGAGATCTTCTAGAAAGATTA CACGAACAGCGATGGAC	*artB*-knockout-plasmid
**artColup_fw**	TGGCTCGAGTTTTTCAGCAAGATCTCG AACCCAAGCGGTAGAG	*artC*-knockout-plasmid
**artColup_rev**	CCTCCACTAGCATTACACTTCGGATTT GGGGCACAGTCTT	*artC*-knockout-plasmid
**artColdown_fw**	TCTTTCCCTAAACTCCCCCCATTCCGTT CCGATCGACTTCGT	*artC*-knockout-plasmid
**artColdown_rev**	TATTGTAGGAGATCTTCTAGAAAGATTG ACTTTGCGGCTCTTCCA	*artC*-knockout-plasmid
**artDolup_fw**	GATGGCTCGAGTTTTTCAGCAAGATTA	*artD*-knockout-plasmid

	CAAAGACAACGGCGGAG	
**artDolup_rev**	CTCCACTAGCATTACACTTGGATCCcgC CCCAAAGGTAACAAGAGAAG	*artD*-knockout-plasmid
**artDoldown_fw**	TTTCCCTAAACTCCCCCACTGCAGaa CGCCCCCTTAGTCTATTTTT	*artD*-knockout-plasmid
**artDoldown_rev**	GTAGGAGATCTTCTAGAAAGATTCTTG CTCTCCTTTTTCTGTC	*artD*-knockout-plasmid
**H2b-FW**	GAAGAAGGCAGGAAAGAAGAC	House-keeping gene
**H2b-Rev**	TTTGGCAGACGAGGAAGAG	real-time PCR
**2597-Fw**	CTGTCATCAACACCCTGAACC	2597 real-time PCR
**2597-Rev**	TCGCTTCTCTCGCTCTATCC	2597 real-time PCR
**2598-Fw**	GACCCCGCTACATTGGAAAC	2598 real-time PCR
**2598-Rev**	TACCCTTCACTCCCTCCTCTAC	2598 real-time PCR
**2599-Fw**	CAACCATACCTCCTCCAACCTC	2599 real-time PCR
**2599-Rev**	ACTTCCATCGCTCTGACCTG	2599 real-time PCR
**artA-fw**	AACATCTCATCTCCCTCACC	*artA* real-time PCR
**artA-Rev**	AACTCCCATCAGTCAAGCC	*artA* real-time PCR
**artB-FW**	GACCCCAAGATTGAAAGAGCC	*artB* real-time PCR
**artB-Rev**	CTACCACCTTCCAACTGAACAC	*artB* real-time PCR
**artC -fw**	GTCGTTCTACTTCGATCTCG	*artC* real-time PCR
**artC -fw**	GTACTCTATACCCTTTGTCG	*artC* real-time PCR
**artD-Fw**	CGAAACTACTTGATGAGCCTCC	*artD* real-time PCR
**artD-Rev**	TCCGCTTTGCCACTTGTTTAC	*artD* real-time PCR
**2605-Fw**	GTTCTCCAGACCAAGTTCAGC	2605 real-time PCR
**2605-Rev**	TCCCCATCTATTTCCCACACC	2605 real-time PCR
**artE-FW**	CGATGATCCCGAAGCACAAG	*artE* real-time PCR
**artE-REV**	CGCAAGAAGGAAGATGGCAC	*artE* real-time PCR
**pjetBB_rekom_rev**	ATCTTGCTGAAAAACTCGAGC	*artA* re-complementation
**trpcP_for**	AAGTGTAATGCTAGTGGAGGT	*artA* re-complementation

**PksA_LB_recomp_for**	GATGGCTCGAGTTTTTCAGCAAGATTCTCATGACAAGGTTGCGCT	*artA* re-complementation
**PksA_recomp _rev**	CGCTCTCGGAAAACTACACT	*artA* re-complementation
**PksA_recomp_for**	AGTGTAGTTTTCCGAGAGCG	*artA* re-complementation
**PksA_RB_recomp_rev**	GTTGACCTCCACTAGCATTACACTTACATAACCGAACACCAAACAG	*artA* re-complementation
**pksA_Ao_for**	AAGAGTCAGTCAGTCTTAATATGACTTCTACGCCAGCGA	Heterologous expression of *artA* in A.o
**PksA_recomp_for**	AGTGTAGTTTTCCGAGAGCG	Heterologous expression of *artA* in A.o
**PksA_recomp _rev**	CGCTCTCGGAAAACTACACT	Heterologous expression of *artA* in A.o
**pksA_Ao_rev**	AGATCGACTGACTGACTTTATCAGACCTTCTCTTCAAACCA	Heterologous expression of *artA* in A.o
**artA(p)-h2b-fw**	CTCTTTCCCTAAACTCCCCCCACCTCAACGAACCGCTTACA	fluorescent signal of *artA*
**artA(p)-h2b-rev**	CGGCGGCTTTTGGTGGCATGGCAGGCCAAAGAAGTCAAG	fluorescent signal of *artA*
**artC(p)-h2b-fw**	CTCTTTCCCTAAACTCCCCCCAAGGCAAAAGTCGTAACCATATC	fluorescent signal of *artC*
**artC(p)-h2b-rev**	CGGCGGCGGCTTTTGGTGGCATTTTGTGAATTGTTTAAAATTAA	fluorescent signal of *artC*
**artB(p)-h2b-fw**	CTCTTTCCCTAAACTCCCCCCAAAGGATGAGAGGAAAGCGG	fluorescent signal of *artB*
**artB(p)-h2b-rev**	GCGGCGGCTTTTGGTGGCATTTTTTGCGCATACAAAAGAGAG	fluorescent signal of *artB*
**artB(p)-h2b-rev**	GCGGCGGCTTTTGGTGGCATTTTTTGCGCATACAAAAGAGAG	fluorescent signal of *artB*
**ArtAend_pjet_ol_fo**	GGATGGCTCGAGTTTTTCAGCAAGATC	ArtA-GFP tag *in locus*

**r**	CAACGACGATTCAGCTATC	
**ArtAend_gfp_ol_rev**	TACTTACCTCACCCTTGGAAACCATGACCTTCTCTTCAAACCATTTAA	ArtA-GFP tag *in locus*
**Gfp_ArtAend_ol_for**	GGTTAAATGGTTTGAAGAGAAGGTCATGGTTTCCAAGGGTGAGGT	ArtA-GFP tag *in locus*
**Gfp_H_ol_rev**	GTTGACCTCCACTAGCATTACACTTCTAAGCGGCCGCTTTGTAAA	ArtA-GFP tag *in locus*
**H_Gfp_ol_for**	TGAACTTTACAAAGCGGCCGCTTAGAAGTGTAATGCTAGTGGAGGT	ArtA-GFP tag *in locus*
**H-ArtARB-ol-rev**	AGAAAAACCCATACCAACACTTCCCTGGGGGGAGTTTAGGGAAA	ArtA-GFP tag *in locus*
**ArtARB_hph_ol_for**	ATGCTCTTTCCCTAAACTCCCCCCAGGGAAGTGTTGGTATGGGTT	ArtA-GFP tag *in locus*
**ArtARB_pjet_ol_rev**	ATTGTAGGAGATCTTCTAGAAAGATCAGTATCACCAATTCTCATCTG	ArtA-GFP tag *in locus*
**ArtBend_pJet_ol_for**	GATGGCTCGAGTTTTTCAGCAAGATCGCGATATGGAGCGATTGTA	ArtB-mCherry tag *in locus*
**ArtBend_mCherry_ol_rev**	tacttacCTCGCCCTTGCTTACCATTATCTTGGCCTCAACTTGCC	ArtB-mCherry tag *in locus*
**mCherry_artBend_ol_for**	CACGGGGCAAGTTGAGGCCAAGATAATGGTAAGCAAGGGCGAG	ArtB-mCherry tag *in locus*
**mCherry_H_ol_rev**	GTTGACCTCCACTAGCATTACACTTttaTTTGTAGAGTTCATCCATTC	ArtB-mCherry tag *in locus*
**H_mCherry_ol_for**	TGGAATGGATGAACTCTACAAAtaaAAGTGTAATGCTAGTGGAGGT	ArtB-mCherry tag *in locus*
**H_artBRB_ol_rev**	CAGTACAAGATATCGGCAAAACACATGGGGGGAGTTTAGGGAAA	ArtB-mCherry tag *in locus*
**ArtBRB_H_ol_for**	ATGCTCTTTCCCTAAACTCCCCCCATGTGTTTTGCCGATATCTTGTA	ArtB-mCherry tag *in locus*

**ArtBRB_pJet_ol_rev**	ATTGTAGGAGATCTTCTAGAAAGATAC GAGACTACACGAACAGC	ArtB-mCherry tag *in locus*

Reference

1 Youssar, L. *et al.* Intercellular communication is required for trap formation in the nematode-trapping fungus *Duddingtonia flagrans*. *PLoS Genet* **15**, e1008029 (2019).
